# Can there be calm during a cytokine storm? Immune checkpoint pathways affecting the severity of COVID-19 disease

**DOI:** 10.3389/fmicb.2024.1508423

**Published:** 2024-12-23

**Authors:** Matyas Meggyes, David U. Nagy, Ildiko Toth, Timoteus Feik, Agnes Peterfalvi, Beata Polgar, David Sipos, Agnes Kemeny, Laszlo Szereday

**Affiliations:** ^1^Department of Medical Microbiology and Immunology, Medical School, University of Pecs, Pecs, Hungary; ^2^Janos Szentagothai Research Centre, Pecs, Hungary; ^3^Institute of Geobotany/Plant Ecology, Martin-Luther-University, Halle, Germany; ^4^Department of Anesthesiology and Intensive Therapy, Medical School, University of Pecs, Pecs, Hungary; ^5^Department of Laboratory Medicine, Medical School, University of Pecs, Pecs, Hungary; ^6^Division of Infectious Diseases, 1^st^ Department of Medicine, Medical School, University of Pecs, Pecs, Hungary; ^7^Department of Pharmacology and Pharmacotherapy, Medical School, University of Pecs, Pecs, Hungary; ^8^Department of Medical Biology and Central Electron Microscopic Laboratory, Medical School, University of Pecs, Pecs, Hungary

**Keywords:** SARS-CoV-2, cytokine storm, immune checkpoints, COVID-19, immune dysregulation

## Abstract

**Introduction:**

The COVID-19 pandemic has become a global health crisis, eliciting varying severity in infected individuals. This study aimed to explore the immune profiles between moderate and severe COVID-19 patients experiencing a cytokine storm and their association with mortality. This study highlights the role of PD-1/PD-L1 and the TIGIT/CD226/CD155/CD112 pathways in COVID-19 patients.

**Methods:**

We performed a study using flow cytometry to compare the phenotypic and functional characteristics of peripheral blood mononuclear cells in patients with moderate or severe disease and healthy individuals. Soluble immune checkpoint molecule and ligand levels were measured by Luminex.

**Results:**

Severe patients show reduced CD8+ T cell frequency, hyperactivation of CD8+ T, NK and NKT cells with concurrent upregulation of immune checkpoint ligands in monocytes. TIGIT expression by CD8+ T and NK cells and PD-1 by NKT cells suggest a spectrum of immune dysfunction, encompassing both hyperactivation and features of exhaustion. This dual phenomenon likely contributes to the impaired viral clearance and the exacerbation of inflammation characteristic of severe disease. Additionally, the study suggests that increased activation and cytotoxicity of NK cells may be associated with fatal outcomes in severe COVID-19 infection.

**Conclusion:**

These findings shed light on the intricate immune response regulation in COVID-19, emphasizing the importance of immune checkpoint pathways and activation signatures in disease severity. A novel aspect of this study is that it includes only COVID-19 patients experiencing cytokine storms, allowing for a focused analysis of immune dysregulation during this critical phase of the disease.

## Introduction

The coronavirus disease 2019 (COVID-19) pandemic, caused by severe acute respiratory syndrome coronavirus 2 (SARS-CoV-2), continues to pose a significant global health threat, leading to substantial morbidity and mortality. While most infected individuals are either asymptomatic or exhibit mild to moderate symptoms, some patients develop severe and potentially fatal infections (Chen et al., 2020; Shahbaz et al., 2021). SARS-CoV-2 infection, characterized by lymphopenia and abnormal T-cell responses, causes immune dysregulation that leads to uncontrolled inflammation, commonly referred to as a cytokine storm (Al-Mterin et al., 2022). Cytokine storms contribute significantly to COVID-19 severity and mortality (Hu et al., 2021; Herr et al., 2021; Cappanera et al., 2021; Vanderbeke et al., 2021).

While an effective immune response is crucial for the elimination of SARS-CoV-2 and the resolution of COVID-19, an excessive immune response has been proposed as a contributing factor to the development of severe cases with high mortality rates (Chen et al., 2020; Jouan et al., 2020; Parrot et al., 2020). However, the transition from innate to adaptive immune responses is critical in determining the clinical implications of COVID-19 infections. Initial responses are often protective, whereas the latter - especially when excessive - leads to reduced viral clearance and lower survival rates. The observed tissue damage in acute COVID-19 infections primarily stems from the hyperreactivity of lymphocyte responses (Al-Mterin et al., 2022).

An overactive immune response against microbial antigens may further augment the immune pathogenesis and lead to severe disease manifestations. Therefore, the upregulation of inhibitory immune checkpoint molecules may be crucial in minimizing immunopathology. In COVID-19, overexpression of coinhibitory receptors like TIM-3 and PD-1 may indicate T-cell exhaustion or hyperactivation (Mishra et al., 2022; Gambichler et al., 2024). However, the specific factors that determine whether an individual will develop the severe form of the disease remain a subject of scientific debate.

There is ample evidence documenting the activation of the immune system at all levels in response to SARS-CoV-2. Monocytes constitute approximately 10–15% of human peripheral blood mononuclear cells (PBMCs) (Kapellos et al., 2019). They represent significant players during the first wave of the innate immune response and can be broadly classified into three populations: classical (CD14+ CD16-), intermediate (CD14+ CD16+), and non-classical (CD14- CD16+) cells. They participate in antigen presentation and activate the adaptive immune response cells during viral infections, including CD4+ and CD8+ T lymphocytes (Affandi et al., 2021; Jakubzick et al., 2017).

NK cells are innate lymphocytes possessing potent cytotoxic machinery, including the secretion of perforin and granzymes, facilitating the rapid elimination of infected cells within a few hours (Vivier et al., 2008). Human NK cells can be classified as CD56dim and CD56bright with distinct effector functions. Activating NK cells during an infection necessitates precise regulation to ensure the elimination of infected cells while preventing the onset of a hyper-inflammatory syndrome.

Unconventional T cell population includes NKT cells, contributing to mucosal homeostasis, inflammatory response, and antimicrobial immunity (Jouan et al., 2020). Due to their versatile functions, these cells may serve as essential players in the immunopathology induced by SARS-CoV-2. Primarily resident in mucosal tissues, including the lungs, they can rapidly respond upon activation by producing inflammatory cytokines and exerting cytotoxic activity. Moreover, they can fine-tune the host immune response’s intensity and quality, shaping the adaptive response’s magnitude.

The immune system relies equally on innate and adaptive immune cells to combat viral infections. CD4+ and CD8+ T cells play significant roles in the body’s defense against viral infections. CD4+ and CD8+ T cells are critical in coordinating and executing immune responses against SARS-CoV-2. CD8+ T cells, also known as cytotoxic T cells, are primarily responsible for directly killing virus-infected cells. They can recognize and bind to specific viral antigens presented by infected cells, releasing cytotoxic molecules such as perforin and granzyme and inducing apoptosis of the infected cells.

The PD-1/PD-L1 axis regulates immune responses and contributes to COVID-19 pathogenesis (Aghbash et al., 2021; Ronchi et al., 2022; Rha et al., 2021). The TIGIT/CD226/CD155/CD112 pathway also has a critical role in maintaining immunological tolerance, but literature about their function in the context of SARS-CoV-2 infection is currently lacking.

COVID-19 severity has been associated with immune system dysregulation, but the specific mechanisms remain unclear. Identifying specific immune-cell subpopulations expressing IC receptors or ligands in severe versus moderate/mild cases is crucial. Comprehensive investigations are currently limited to exploring the co-expression and interplay of multiple IC receptors or ligands on specific immune-cell subpopulations in COVID-19 patients. While a significant body of literature exists on SARS-CoV-2 research, studies focusing specifically on how immune cells expressing immune checkpoint molecules and ligands respond to this novel virus still need to be made available.

The primary objective of this study is to investigate the differential immune profiles of patients with moderate and severe COVID-19, focusing on the mechanisms underlying cytokine storms and their association with disease severity and mortality. A novel aspect of this study is that it includes only COVID-19 patients experiencing cytokine storms, allowing for a focused analysis of the immune dysregulation during this critical phase of the disease.

Specifically, we aim to examine the role of immune checkpoint pathways, particularly the PD-1/PD-L1 and TIGIT/CD226/CD155/CD112 axes, in modulating the immune responses in these patients. By comparing the phenotypic and functional characteristics of peripheral blood mononuclear cells and the expression levels of immune checkpoint molecules and ligands, we seek to elucidate how these pathways contribute to COVID-19 severity and identify potential targets for therapeutic intervention.

## Materials and methods

### Clinical study design (demographics and basic characteristics of severe and moderate COVID-19 patients)

In this clinical study, we established a cohort of 35 patients admitted to the University of Pécs, Pécs, Hungary, during the third pandemic wave, specifically when the Delta-variant was predominant. The patient recruitment period for the cohort was from 23 April 2021 to 7 December 2021. Detailed information about the cohort is presented in Table 1. Eighteen patients with COVID-19 were admitted to the infectious disease unit (IDU), representing cases with moderate severity. Seventeen patients were admitted to one of the intensive care units (ICU), indicating severe cases requiring more intensive medical care. It is worth noting that the ICU group had a significant mortality rate of 64%. RT-PCR tests proving SARS-CoV-2 infection on admission were performed by LightMix^®^ Modular E- and N-gene kits on Cobas Z 480 PCR platform (Roche Diagnostics GmbH, Mannheim, Germany). Patients were recruited based on Cappanera et al. (2021) paper about a quick COVID-19 cytokine storm score. This scoring system includes the following laboratory values: lymphocyte count <1,000/mm^3^, D-dimer >1,000 ng/mL, LDH >300 IU/L, Ferritin >500 ng/mL, and CRP >10 mg/dL. The disease severity and clinical prognosis of the coronavirus-infected patients were evaluated using (1) sequential organ failure assessment (SOFA) score, (2) simplified acute physiology score (SAPS) and (3) Computed tomography (CT) score of lung involvement (Table 2).

**Table 1:** Demographic characteristics and comorbidities of patients on admission.

Variables	Healthy controls (*n* = 14)	IDU patients (*n* = 18)	ICU patients (*n* = 17)	*p*-value
Age, mean, years (range)	44 (16–73)	52.2 (19–66)	66 (46–86)	**<0.05 HC vs. ICU <0.05 IDU vs. ICU**
Females/males	6 / 8	6 / 12	6 / 11	NS
Obesity (BMI, median)	NA	27.78 (19.11, 45.2)	29.4 (21.48, 71.78)	NS
Comorbidities				
Hypertension (*n*, %)	0	6 (33.3%)	14 (82.4%)	**<0.01 ICU vs. IDU**
Heart disease (*n*, %)	0	0	3 (17.6%)	NS
Chronic lung disease (*n*, %)	0	1 (5.5%)	0	NS
Chronic kidney disease (*n*, %)	0	0	2 (11.7%)	NS
Diabetes (*n*, %)	0	2 (11.1%)	6 (35.3%)	NS
Days elapsed between the onset of symptoms and admission	–	6.7 ± 3.6	5.4 ± 4.1	NS

Data are presented as mean ± standard deviation, median (minimum, maximum) or numbers and percentages. Differences were considered significant when the P value was equal to or less than 0.05. Comparison of IDU vs. ICU patients on the day of admission vs. healthy controls. ICU, Intensive care unit, IDU, infectious disease unit, HC, healthy control, BMI, body mass index; NS, not significant; NA, data not available. Significant results are presented in bold.

**Table 2:** Laboratory and clinical characteristics of ICU and IDU patients.

Laboratory parameters	IDU patients (*n* = 18)	ICU patients (*n* = 17)	*p*-value
Lymphocyte count (cells/mm^3^)	0.8 ± 0.17	0.66 ± 0.21	**<0.01**
LDH (U/L)	431 ± 232	926 ± 476	**<0.001**
D-dimer (ng/ml)	1,027 (228, 24,582)	1,597 (681, 329,800)	NS
Ferritin (ng/ml)	1,035 (42, 6,155)	1,117 (32, 9,667)	NS
CRP (mg/dl)	9.6 ± 6.7	12.8 ± 7.7	NS
IL-6 (pg/ml)	62 (7, 143)	61 (31, 924)	NS
Clinical parameters			
Days of hospitalization	8 ± 5	15 ± 12	**<0.05**
Mortality (*n*, %)	0	11 (64.7%)	
SOFA	2 (0.4)	3 (0, 9)	**<0.05**
SAPS	–	33 (13, 71)	
CT score	11 ± 4.8	15 ± 6.8	**<0.05**
PaO_2_/FiO_2_ on admission	194 (32, 410)	67 (43, 388)	**<0.05**
Mechanical ventilation (days)	–	4 (0, 19)	
Kidney replacement therapy (*n*, %)	–	3 (17%)	
Vasopressor therapy (*n*, %)	–	13 (76%)	

Data are presented as mean ± standard deviation or median (minimum, maximum). Differences were considered significant when the *P* value was equal to or less than 0.05. ICU, Intensive care unit; IDU, infectious disease unit; HC, healthy control; LDH, lactate dehydrogenase; CRP, C-reactive protein; IL-6, interleukin-6; SOFA, Sequential organ failure assessment score; SAPS, simplified acute physiology score; CT score, computed tomography score; NS, not significant. Significant results are presented in bold.

The ICU patients were significantly older than those in the other groups, as indicated in Table 1. There was no significant difference in gender distribution among the groups. Hypertension was significantly more frequent among ICU patients compared to patients in IDU. The onset of infection symptoms in both groups was approximately 1 week before hospital admission, with fever, cough and dyspnea emerging as the prevailing and frequently observed clinical manifestation. Out of the 35 patients included in the study, 24 successfully recovered and were discharged, while 11, all from the severe group, died while hospitalized. Among the deceased patients, 5 were female and aged 45 years or older. The median age of deceased cases was 67.6 years. Moreover, ICU patients were also hospitalized for significantly longer.

For comparative purposes, blood samples were collected from 14 healthy individuals who were not infected, unvaccinated, non-hospitalized and symptom-free during the pandemic. These individuals tested negative for anti-SARS-CoV-2 spike and nucleocapsid antibodies, as measured using the Roche Cobas automated clinical chemistry analyzer from Roche, Switzerland.

Among the laboratory values measured upon admission, lymphocyte count and LDH exhibited a significant difference between the two groups. ICU patients demonstrated the presence of ARDS upon admission and concurrently had significantly worse CT scores, which described the number of infected lung lobes (Prakash et al., 2023). Only ICU patients needed organ support therapies like mechanical ventilation, kidney replacement, and vasopressor therapy. ICU patients had significantly more extended hospital stays than the IDU group.

Upon admission, peripheral venous blood samples were collected from all patients before initiating any pharmaceutical treatment. A closed-system blood collection procedure was employed using standard plain and heparinized blood collection tubes (Greiner, Austria). Written informed consent was obtained from all patients or their legal representatives before inclusion in the study. Patients were treated based on the local hospital protocol; our study did not influence their therapy. The research protocol was approved by the local Ethics Committee aligned with the Medical School, University of Pécs, Pécs, Hungary (Ethical registration number: 8759-PTE/2021). The study protocol conforms to the ethical guidelines of the 2013 revised version of the Declaration of Helsinki.

### Lymphocyte separation, cryopreservation, and thawing

Peripheral blood mononuclear cells (PBMC) were separated from heparinized venous blood on Ficoll-Paque (GE-Healthcare, USA) density gradient. The PBMC fraction was washed in complete Rosewell Park Memorial Institute medium 1,640 (RPMI, Lonza, Switzerland) supplemented with 10% fetal calf serum (FCS, Lonza, Switzerland). The cells were counted and centrifuged, and for cryoprotection, they were resuspended in inactivated human AB serum containing 10% DMSO (Sigma-Aldrich, USA). The cryopreserved cells were stored at −80°C in a mechanical freezer for further investigation. On the day of examinations, the cryopreserved samples were thawed in a 37°C water bath and resuspended in RPMI 1640 medium. To eliminate any residual DMSO content, the cells were washed twice.

### Flow cytometric measurement

Before surface labeling of thawed PBMCs with a combination of fluorochrome-conjugated monoclonal antibodies, Fc receptor-expressing monocytes were blocked with Human TruStain FcX Blocking Solution (Biolegend, USA) for 10 min. For flow cytometric labeling, a combination of fluorochrome-conjugated monoclonal antibodies (Supplementary Table S1) was added to 10^6^ PBMCs for 30 min at room temperature in complete darkness. Following a washing step, the cells were resuspended in 300 μl PBS (BioSera, France) containing 1% paraformaldehyde (PFA) and stored at 4°C in complete darkness until analysis using FACS. This study also quantified the relative expression levels of various cell surface markers using mean fluorescent intensity (MFI) measurements. Flow cytometric measurements were conducted using a BD FACS Canto II flow cytometer (BD Immunocytometry Systems, Belgium) and the BD FACS Diva V6 software (BD Biosciences, USA) for data acquisition. Flow cytometric data were analyzed using FCS Express V4 software (*De Novo* Software, USA).

### Intracellular staining

Following surface labeling, cells were washed with PBS and fixed using 4% PFA for 10 min at room temperature in complete darkness. Next, the cells were washed with PBS and permeabilized by incubating them with a 1:10 dilution of FACS Permeabilizing Solution 2 (BD Biosciences, USA) for 10 min at room temperature in darkness. Then, the samples were washed and incubated with anti-human granzyme A, granzyme B, and perforin antibodies for 30 min at room temperature in complete darkness. The samples were washed with PBS, fixed using 1% PFA, and stored at 4°C in the darkness until FACS analysis.

### Cytotoxic activity measurement

Isolated PBMCs were incubated in the presence of FITC-conjugated anti-human CD107a monoclonal antibody in RPMI 1640 medium containing 10% fetal bovine serum, penicillin, and streptomycin, ionomycin (Sigma–Aldrich, USA), and phorbol myristate acetate (Sigma–Aldrich, USA) for 4 h at 37°C. Subsequently, the samples were washed, resuspended in PBS, and stained with a combination of antibodies for 30 min at room temperature in the dark to identify the CD8+ T and NK cell subpopulations. Finally, the cells were washed in PBS, fixed with 1% PFA and evaluated by FACS.

### Measurement of the concentration of 8 distinct protein markers from serum samples using Luminex xMAP technology

Human peripheral blood samples were collected from healthy controls and all patients and were allowed to clot for 25 min before centrifugation at 1,000 x g for 10 min at 20–25°C. Serum fractions were collected, pooled and stored at −80°C before running the assay. Luminex xMAP technology was used to determine the protein concentrations of PD-1, PD-L1, Granzyme B, CD226, nectine-2, perforin, PVR (CD155); and Granzyme A cytokines/chemokines performing Milliplex Human Immuno-Oncology Checkpoint Protein Panel 1, 2 or Milliplex Human CD8+ T-Cell Magnetic Bead panels, respectively (Merck KGaA, Darmstadt, Germany) according to the instructions of the manufacturer. Briefly, all samples were thawed and tested undiluted in a blind fashion and duplicated. 25 μL volume of each sample, standard and control was added to a 96-well plate (provided with the kit) containing 25 μL of capture antibody coated bead sets, each internally color-coded with fluorescent dyes. Following overnight incubation, a biotinylated detection antibody mixture and streptavidin-PE were added to the plate after appropriate washing steps. After the last washing step, 150 μL drive fluid was added to the wells, and the plate was incubated for an additional 5 min on a shaker and immediately read on the Luminex MAGPIX^®^ instrument (Merck KGaA, Darmstadt, Germany). Luminex xPonent 4.2 software was used for data acquisition. Five-PL regression curves were generated to plot the standard curves for all analytes by the Belysa v1.1 (Merck Millipore, Darmstadt, Germany) software calculating with bead median fluorescence intensity values. Results are given in pg./ml or, in the case of Granzyme A, in ng/ml.

### Statistical analyses

Two-Way-ANOVA was applied for statistical significance testing in R, version 4.2.2 (R Core Team, 2022) testing the interactive effect of group × CD112, group × CD107a, survival × CD107a and group × survival on the measured response variables. One-way ANOVA was applied to test the main effect of the group on the measured response variables. Linear regressions were used to test the relationship between the soluble level of circulating factors (PD-1, PD-L1 and perforin) and the relative or intracellular expression of these molecules. Response variables were log_e_ transformed before analysis. Decisions on the transformation of variables depended on visual inspection of “model-checking plots” in R for the models with transformed vs. untransformed variables. These plots allow checking assumptions about the normality of residuals and variance homogeneity. For pair-wise comparisons of ANOVA tests, Tukey posthoc tests were conducted to compare combinations to each other (R Core Team, 2022). Given that ICU patients were significantly older than those in the other groups, we analyzed our data by including age as an additive covariate to enhance the accuracy of our statistical evaluation (see Table 2).

## Results

### Altered frequency of innate and adaptive immune cells in the blood of patients with severe and moderate COVID-19 disease

Our study aimed to analyze the survival outcomes of hospitalized COVID-19 patients and identify its associated factors. We found that all the patients who did not survive belonged to the severe group receiving ICU care, while every individual in the moderate group treated at IDU survived. We initiated our investigation into the immune cell response in SARS-CoV-2 infection by examining the frequency of immune cells in peripheral blood samples obtained from patients with severe (ICU patients) and moderate (IDU patients) COVID-19 and uninfected healthy controls (Table 3).

**Table 3:** Phenotype analysis of peripheral blood mononuclear cells.

	Gate	Healthy controls (*n* = 14)	IDU patients (*n* = 18)	ICU patients (*n* = 17)	*p*-value
CD3+ T cells	Lymphogate	57.10 ± 10.63	56.13 ± 9.93	50.38 ± 11.97	NS
CD4+ T cells	Lymphogate	30.20 ± 13.88	29.50 ± 10.93	32.14 ± 13.64	NS
CD4+ T cells in CD3+ T cells	Lymphogate	50.70 ± 16.61	52.66 ± 16.34	62.21 ± 14.75	NS
CD8+ T cells	Lymphogate	20.82 ± 4.60	20.57 ± 8.52	14.88 ± 7.41	**<0.05 HC vs. ICU** **<0.05 IDU vs. ICU**
CD8+ T cells in CD3+ T cells	Lymphogate	37.86 ± 11.31	36.80 ± 14.01	30.81 ± 15.39	NS
NK cells	Lymphogate	17.86 ± 8.17	19.99 ± 7.97	21.87 ± 12.54	NS
NK^dim^ cells	Lymphogate	17.30 ± 8.10	19.15 ± 7.75	21.24 ± 12.38	NS
NK^bright^ cells	Lymphogate	0.58 ± 0.33	0.86 ± 0.65	0.66 ± 1.01	NS
NKT-like cells	Lymphogate	7.98 ± 6.69	7.62 ± 7.65	5.47 ± 4.19	NS
Classical monocytes	Monocyte	94.98 ± 2.65	98.07 ± 1.27	96.85 ± 0.92	**<0.05 HC vs. IDU**
Intermediate monocytes	Monocyte	1.31 ± 0.94	1.25 ± 0.93	1.95 ± 4.35	NS
Non-classical monocytes	Monocyte	3.61 ± 1.95	0.37 ± 0.36	0.74 ± 0.79	**<0.01 HC vs. IDU <0.01 HC vs. ICU**

The results were expressed in percentages of cells as the mean value ± standard deviation of the mean (SD). Differences were considered significant when the P value was equal to or less than 0.05. ICU, Intensive care unit; IDU, infectious disease unit; HC, healthy control; NS, not significant. Significant results are presented in bold.

Using a flow cytometric gating strategy (Supplementary Figure S1), we examined the frequency of CD3+, CD4+ and CD8+ T, NK cell subsets and NKT cells in the peripheral blood of COVID-19 patients with moderate and severe disease. CD8+ T cells were significantly reduced in severe cases compared to moderate and healthy groups (Table 3).

We observed a significant increase in the frequency of classical monocytes only in IDU patients with COVID-19 compared to the healthy control group. Conversely, the frequency of the non-classical monocyte subpopulation exhibited a significant decrease in both IDU and ICU patients compared to healthy individuals, consistent with previous studies (Sánchez-Cerrillo et al., 2020; Merad and Martin, 2020a).

### Altered expression of immune checkpoints and ligands by various immune cells in the blood of patients with severe and moderate COVID-19 disease

The immune checkpoint molecule expressions by the investigated immune cell subpopulations were measured using multicolor flow cytometry.

**Figure 1:**
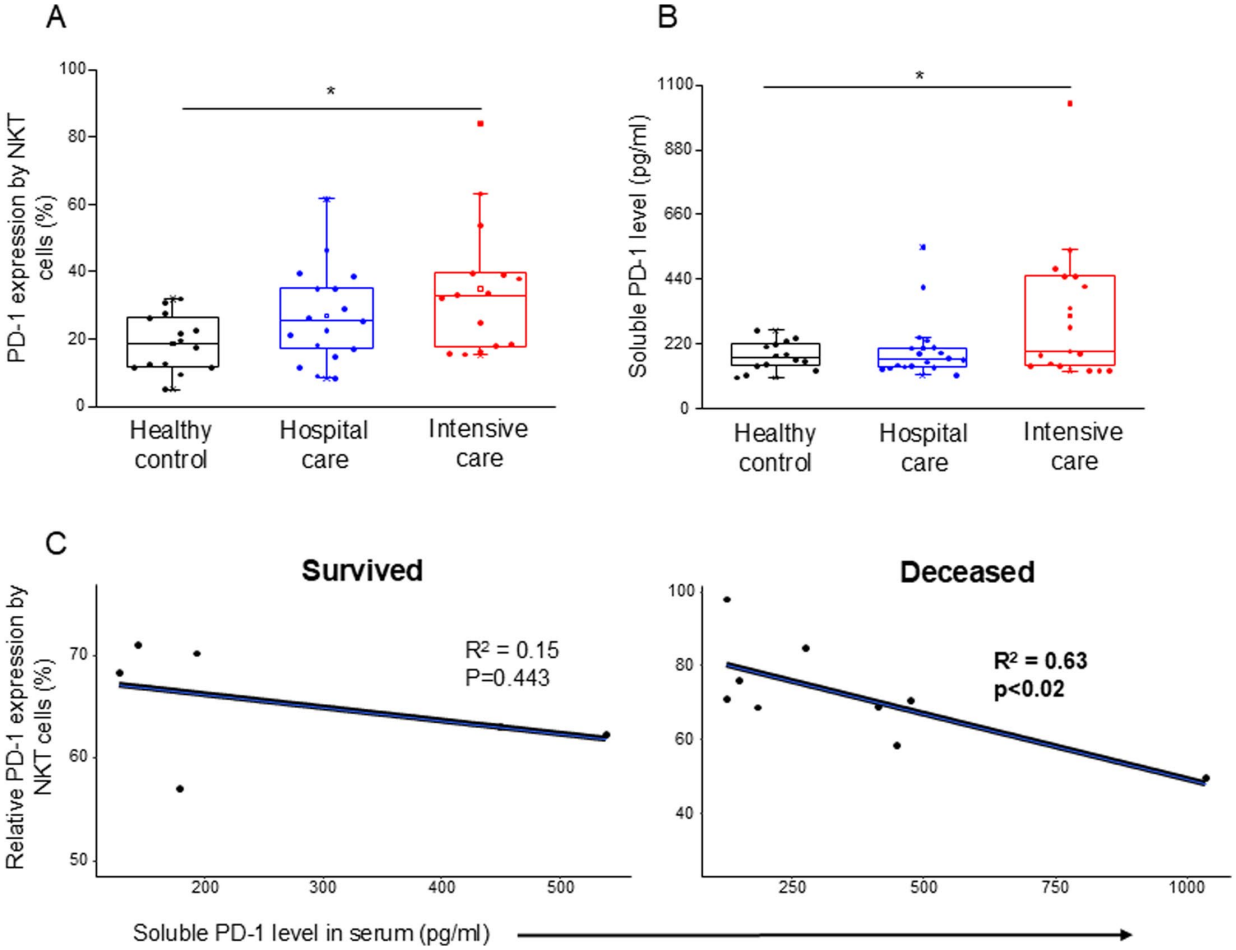
PD-1 expression by NKT cells and soluble level of PD-1 in patients with moderate or severe COVID-19 and healthy controls. The expression of PD-1 receptor by NKT cell population **(A)** and the serum concentration of sPD-1 molecule **(B)** in patients with moderate or severe COVID-19 and healthy controls. The solid bars represent medians, the boxes indicate the interquartile ranges, and the lines show the most extreme observations. Differences were considered statistically significant for *p*-values ≤0.05. Linear regression analyses between the soluble level of PD-1 molecule and the relative PD-1 expression by NKT cells in survived and deceased patients **(C)**. *p* values and coefficients of determination (R^2^) were calculated in R. *p < 0.05.

The inhibitory receptor PD-1 expression (Supplementary Table S2) showed a significant increase by the NKT cells in ICU patients compared to healthy controls (Figure 1A). The level of soluble PD-1 was significantly increased only in severe disease patients compared to healthy individuals (Figure 1B). A significant negative relationship was detected between the relative expression of PD-1 by NKT cells and the level of soluble PD-1 (sPD-1) molecule in deceased ICU patients (Figure 1C).

**Figure 2:**
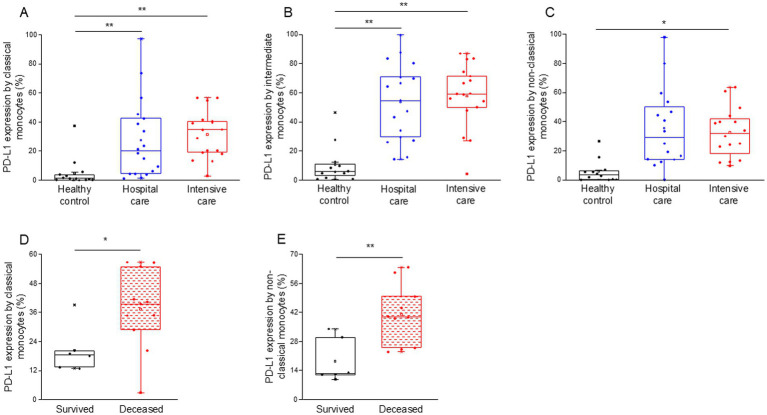
PD-L1 ligand expression by different monocyte subpopulations in patients with moderate or severe COVID-19 and healthy controls. The expression of PD-L1 ligand by classical **(A)**, intermediate **(B)**, and non-classical **(C)** monocytes in patients with moderate or severe COVID-19 and healthy controls. PD-L1 expression by classical **(D)** and non-classical **(E)** monocyte subpopulation in survived and deceased patients. The solid bars represent medians, the boxes indicate the interquartile ranges and the lines show the most extreme observations. Differences were considered statistically significant for p-values ≤0.05. **p < 0.01, *p < 0.05.

Notably, the expression of PD-L1 on classical, intermediate and non-classical monocytes was significantly higher in intensive-care patients with severe disease (ICU) compared to healthy controls (Figures 2A–C). Both classical and intermediate monocytes displayed a significantly increased expression of PD-L1 in hospital care patients with moderate disease (IDU) (Figures 2A,B). Surprisingly, PD-L1 expression by any investigated monocyte cell subpopulations showed no difference between patients with moderate and severe disease (Supplementary Table S3). Remarkably, classical and non-classical monocytes from deceased patients exhibited a significantly higher level of PD-L1 expression compared to patients with severe disease who survived COVID-19 infection (Figures 2D,E).

**Figure 3:**
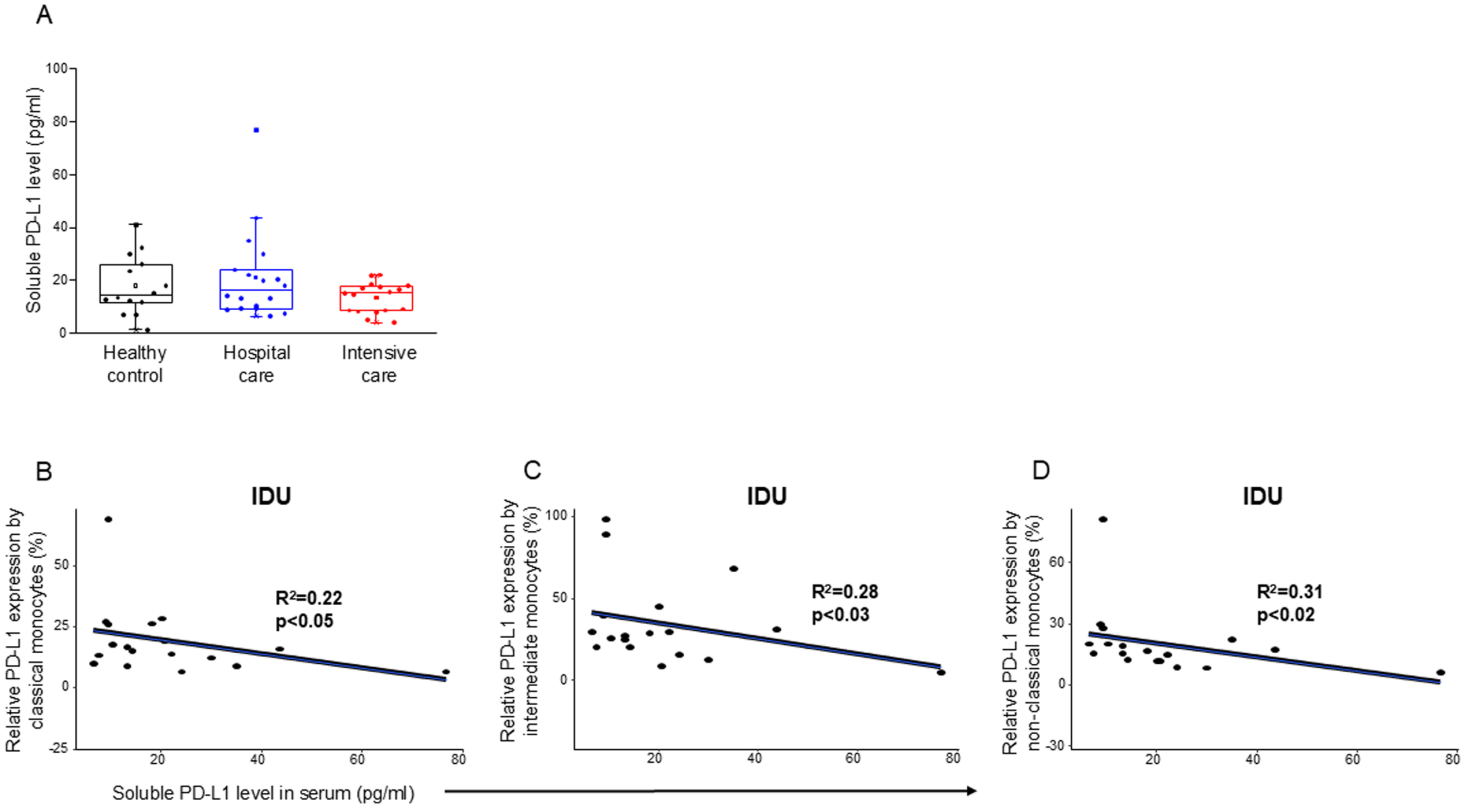
Soluble level of PD-L1 in patients with moderate or severe COVID-19 and healthy controls and regression analyses between the relative PD-L1 expression and soluble PD-L1 level in patients with moderate COVID-19. Serum concentration of soluble PD-L1 **(A)** in patients with moderate or severe COVID-19 and healthy controls. The solid bars represent medians, the boxes indicate the interquartile ranges, and the lines show the most extreme observations. Differences were considered statistically significant for p-values ≤0.05. Linear regression analyses between the level of sPD-L1 molecule and the relative PD-L1 expression by classical **(B)**, intermediate **(C)**, and non-classical **(D)** monocyte subpopulations in moderate COVID-19. p values and coefficients of determination (R^2^) were calculated in R.

Analyzing the level of soluble PD-L1 molecule, we did not find any significant difference among the investigated groups (Table 4) and (Figure 3A). However, a significant negative relationship was detected between the relative PD-L1 expression by all examined monocyte subpopulations and the level of soluble PD-L1 molecule in patients with moderate disease (IDU) (Figures 3B–D). A significant negative relationship was detected between the relative PD-1 expression by CD8+ T cells and the level of soluble PD-1 molecule in patients with moderate disease (IDU) (Supplementary Figure S2).

**Table 4:** Soluble level (pg/ml) of immune checkpoint and cytotoxic molecules.

pg/ml	Healthy controls (*n* = 14)	IDU patients (*n* = 18)	ICU patients (*n* = 17)	*p*-value
PD-1	177.40 ± 48.93	202.86 ± 108.66	315.27 ± 235.33	**<0.05 HC vs. ICU**
PD-L1	18.01 ± 11.20	21.28 ± 17.17	13.56 ± 5.72	NS
CD226	5241.56 ± 6446.11	3434.97 ± 3102.36	2246.97 ± 1981.54	NS
CD155	25306.33 ± 13593.00	36508.44 ± 17912.19	46459.86 ± 17912.19	**<0.01 HC vs. ICU**
CD112	644.15 ± 195.62	1070.89 ± 324.33	1854.79 ± 675.19	**<0.01 HC vs. ICU** **<0.01 IDU vs. ICU <0.05 HC vs. IDU**
Perforin	7418.56 ± 2862.88	5224.87 ± 2288.21	4988.57 ± 3214.66	**<0.052 HC vs. ICU**
Granzyme B	3.31 ± 1.46	15.04 ± 10.77	18.00 ± 20.91	**<0.06 HC vs. IDU** **<0.05 HC vs. ICU**

The results were expressed as the mean value ± SD of the mean. Differences were considered significant when the P value was equal to or less than 0.05. ICU, Intensive care unit; IDU, infectious disease unit; HC, healthy control; NS, not significant. Significant results are presented in bold.

However, no significant differences were detected in the expression level of CD226 (Supplementary Table S4); TIGIT expression by CD8+ T cells from patients with moderate disease was significantly decreased compared to healthy controls and severe patients (Supplementary Table S5).

**Figure 4:**
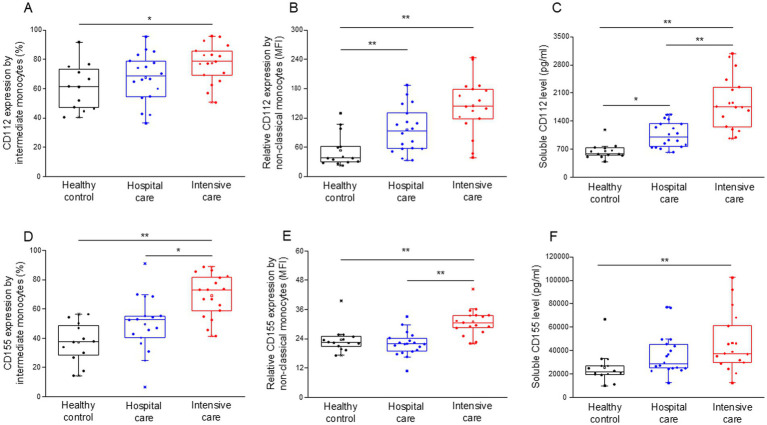
Surface and relative expression of CD112 and CD155 ligand molecules by intermediate and non-classical monocyte subpopulation and the soluble level of CD112 and CD155 molecules in patients with moderate or severe COVID-19 and healthy controls. The surface expression of CD112 by intermediate monocytes **(A)**, the relative expression of CD112 by non-classical monocytes **(B)**, and the serum concentration of soluble CD112 **(C)** in patients with moderate or severe COVID-19 and healthy controls. The surface expression of CD155 by intermediate monocytes **(D)**, the relative expression of CD155 by non-classical monocytes **(E)** and the serum concentration of soluble CD155 **(F)** in patients with moderate or severe COVID-19 and healthy controls. The solid bars represent medians, the boxes indicate the interquartile ranges, and the lines show the most extreme observations. Differences were considered statistically significant for *p*-values ≤0.05. **p < 0.01, *p < 0.05.

The surface expression levels of the CD112 (Supplementary Table S6) and CD155 (Supplementary Table S7) ligands were determined in monocyte subpopulations. The expression of CD112 by intermediate monocytes was significantly elevated in patients with severe disease compared to healthy individuals (Figure 4A). Similarly, the relative expression of CD112 by non-classical monocytes significantly increased in both moderate and severe disease patients compared to healthy individuals (Figure 4B). The level of soluble CD112 increased significantly in both moderate and severe disease patients compared to healthy individuals, with a significant increase in ICU compared to IDU patients (Figure 4C). The expression of CD155 by intermediate monocytes was significantly higher in patients with severe COVID-19 compared to both healthy controls and patients with moderate disease (Figure 4D). The relative expression of CD155 by non-classical monocytes was significantly higher in patients with severe COVID-19 compared to both healthy controls and patients with moderate disease (Figure 4E). The level of soluble CD155 was significantly increased in patients with severe disease compared to healthy individuals (Figure 4F).

An elevated level of serum CD155 (Table 4) was observed in parallel with a significant positive relationship between the relative expression of CD155 by the non-classical monocyte subpopulation and the serum soluble CD155 only in the deceased ICU group (Supplementary Figure S3).

### Altered function of innate and adaptive immune cells in the blood of patients with severe and moderate COVID-19 disease

**Figure 5:**
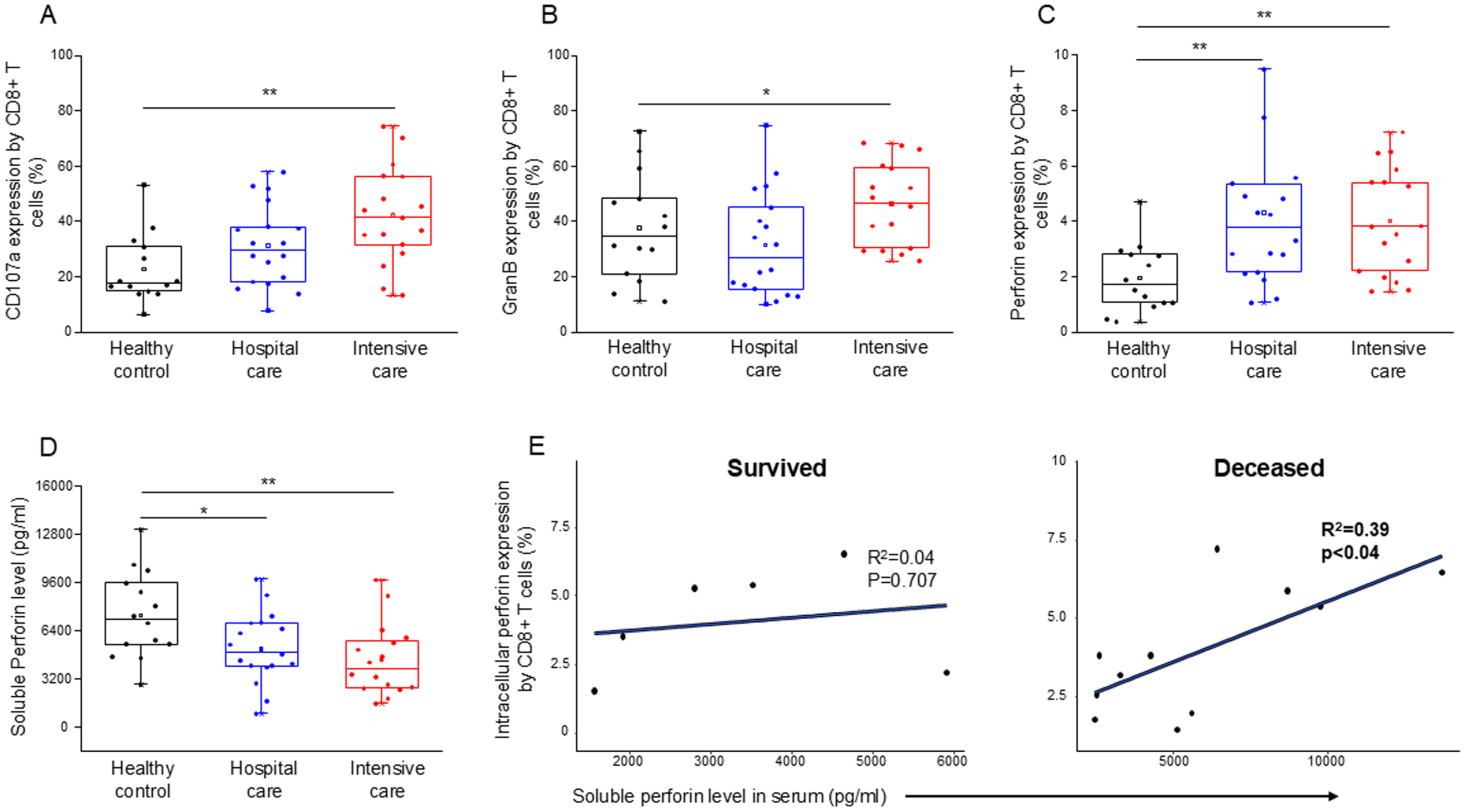
Cytotoxic activity of the CD8+ T cells, soluble level of perforin and the regression analyses between the soluble level of perforin and the intracellular perforin content of CD8+ T cells in patients with moderate or severe COVID-19 and healthy controls. CD107a expression **(A)**, intracellular granzyme B **(B)** and intracellular perforin **(C)** expression by CD8+ T cell. The serum concentration of perforin molecule **(D)** in patients with moderate or severe COVID-19 and healthy controls. The solid bars represent medians, the boxes indicate the interquartile ranges, and the lines show the most extreme observations. Differences were considered statistically significant for p-values ≤0.05. Linear regression analyses between the soluble level of perforin and the intracellular level of perforin by CD8+ T cells in survived and deceased patients **(E)**. *p* values and coefficients of determination (R^2^) were calculated in R. **p < 0.01, *p < 0.05.

CD8+ T cells from patients with severe disease demonstrated a significant increase in cytotoxicity (CD107a expression (Figure 5A)), as well as significantly elevated intracellular levels of granzyme B (Figure 5B) and perforin (Figure 5C), in comparison to healthy controls. Intracellular expression of perforin was also significantly elevated by CD8+ T cells from patients with moderate and severe disease compared to healthy controls (Figure 5C). The level of soluble perforin in the serum significantly decreased in both moderate and severe disease patients compared to healthy individuals (Figure 5D). A significant positive relationship was detected between the intracellular perforin content of CD8+ T cells and the level of soluble perforin only in deceased ICU patients (Figure 5E).

**Figure 6:**
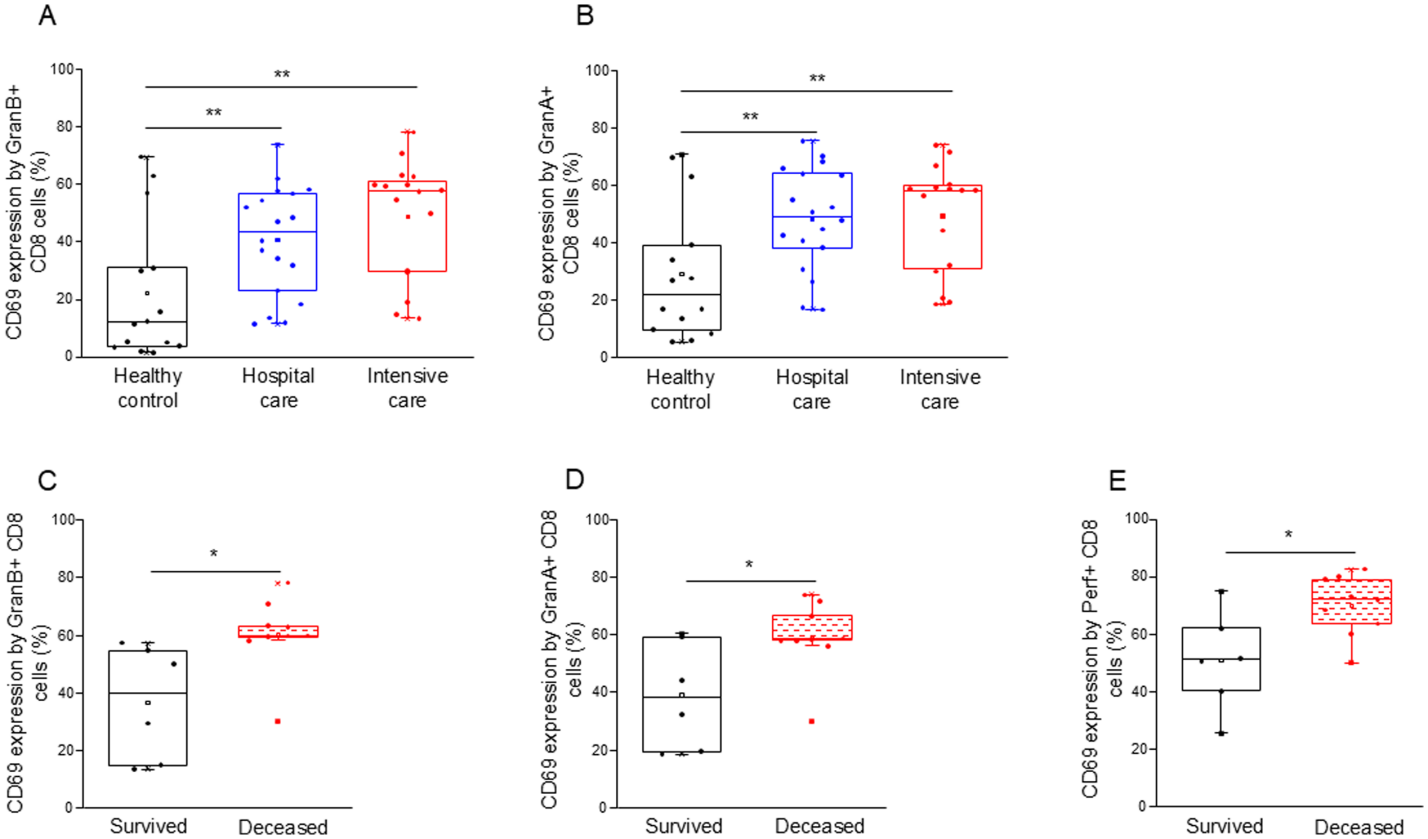
Activation level of the potential cytotoxic CD8+ T cell subpopulation in patients with moderate or severe COVID-19 and healthy controls. CD69 expression by CD8 T cells containing granzyme B **(A)** and granzyme A **(B)** in patients with moderate or severe COVID-19 and healthy controls. CD69 expression by CD8 T cells containing granzyme B **(C)**, granzyme A **(D)**, and perforin **(E)** in survived and deceased patients. The solid bars represent medians, the boxes indicate the interquartile ranges, and the lines show the most extreme observations. Differences were considered statistically significant for *p*-values ≤0.05. **p < 0.01, *p < 0.05.

Both granzyme A and B positive CD8+ T cells were more activated (increased CD69 expression) in all COVID-19 patients compared to the control group (Figures 6A,B). Interestingly, granzyme A, B, and perforin-positive CD8+ T cells from deceased ICU patients were significantly more activated than cells obtained from the survivors (Figures 6C–E).

**Figure 7:**
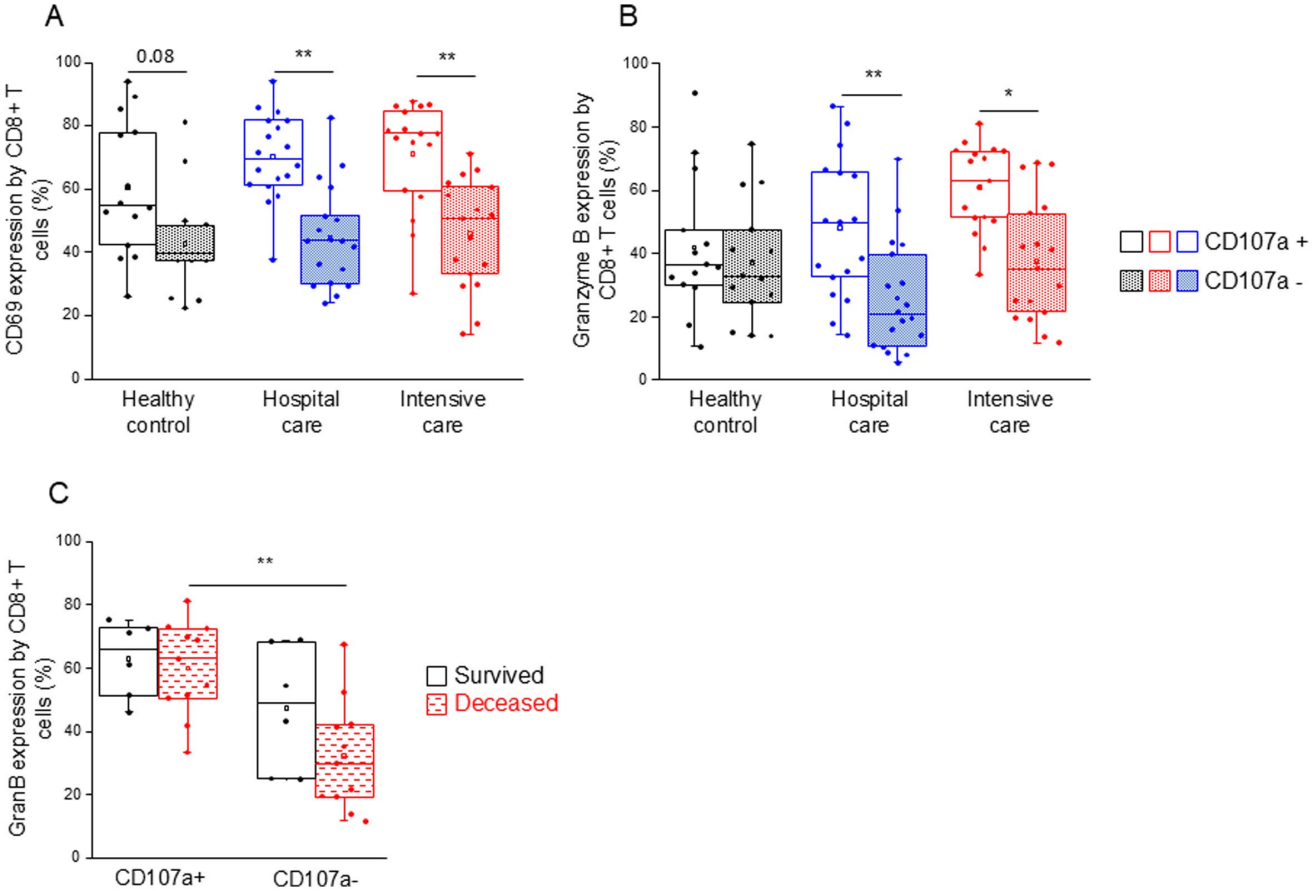
CD69 and granzyme B expression by cytotoxic and non-cytotoxic CD8+ T cell subpopulations in patients with moderate or severe COVID-19 and healthy controls. CD69 **(A)** and granzyme B **(B)** expression by CD107a positive and CD107a negative CD8+ T cell subpopulation in patients with moderate or severe COVID-19 and healthy controls. Granzyme B expression by CD107a positive and CD107a negative CD8+ T cell subpopulation in survived and deceased patients **(C)**. The solid bars represent medians, the boxes indicate the interquartile ranges, and the lines show the most extreme observations. Differences were considered statistically significant for *p*-values ≤0.05. **p < 0.01, *p < 0.05.

CD107a-positive, potentially cytotoxic CD8+ T cells from both patient groups (IDU and ICU) were significantly more activated (CD69 expression (Figure 7A)) and expressed significantly higher levels of intracellular granzyme B compared to non-cytotoxic counterparts (Figure 7B). Granzyme B expression by CD107a-positive CD8+ T cells was significantly increased compared to the CD107a-negative counterparts in deceased patients (Figure 7C). This remarkable change was not observed in patients who survived COVID-19 infection (Figure 7C).

**Figure 8:**
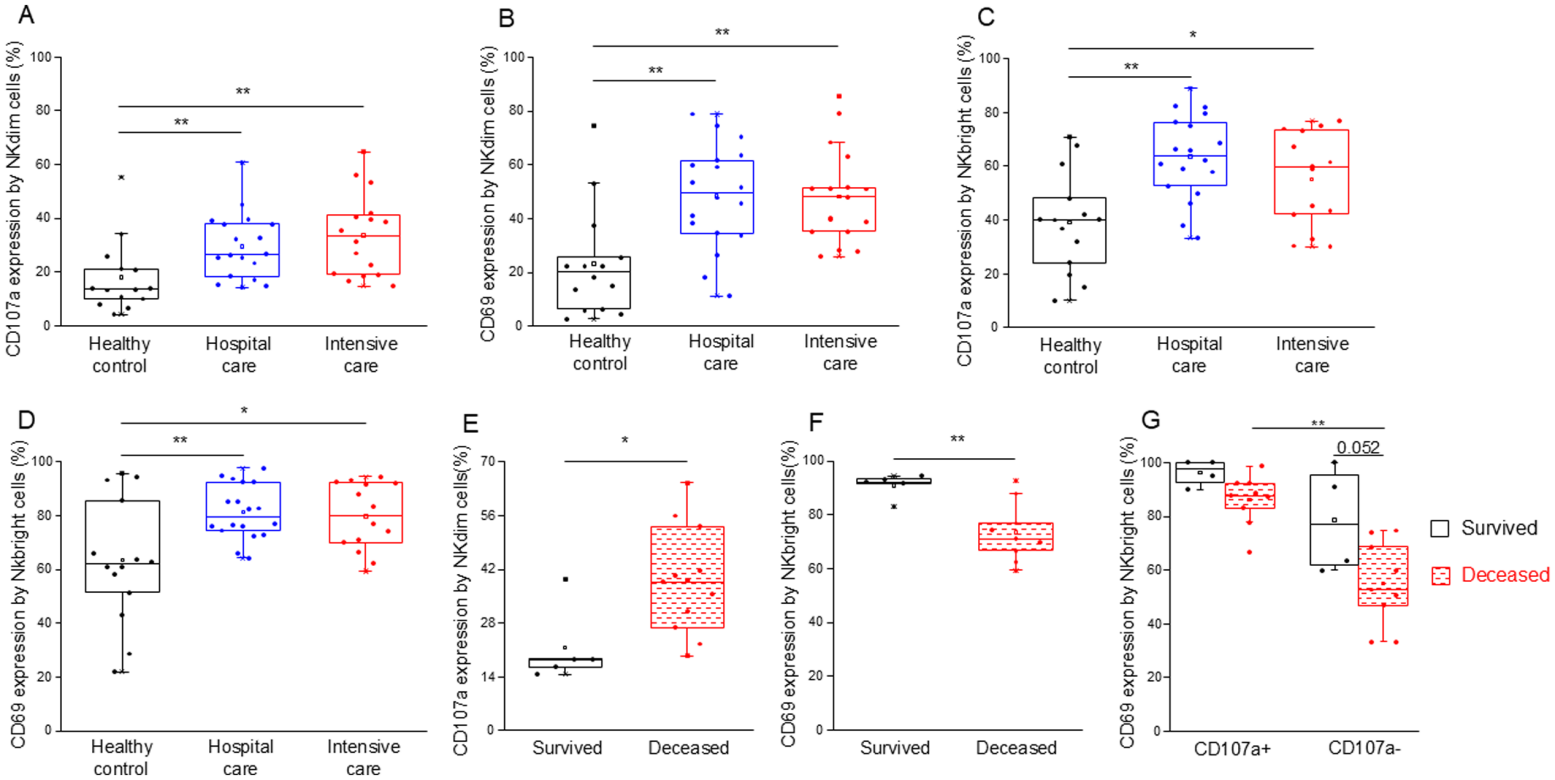
Cytotoxic activity of the different NK cell subpopulations in patients with moderate or severe COVID-19 and healthy controls. CD107a **(A)** and CD69 **(B)** expression by NKdim cells and CD107a **(C)** and CD69 **(D)** expression by NKbright cells in patients with moderate or severe COVID-19 and healthy controls. CD107a expression by NKdim cells **(E)** and CD69 expression by NKbright cells **(F)** in survived and deceased patients. CD69 expression by CD107a positive and negative NKbright cells **(G)** in survived and deceased patients. The solid bars represent medians, the boxes indicate the interquartile ranges, and the lines show the most extreme observations. Differences were considered statistically significant for *p*-values ≤0.05. **p < 0.01, *p < 0.05.

We examined the expression of CD107a and CD69 to assess NK cell activation and cytotoxicity. NKdim (Figures 8A,B) and NKbright (Figures 8C,D) cells showed robust activation and cytotoxicity in both moderate and severe COVID-19 cases.

Upon examining the activity (CD69 expression) of NK cell subpopulations, we observed that both CD107a positive NKdim and NKbright cells were significantly more activated across all investigated groups compared to their less cytotoxic counterparts (Supplementary Figures S4A,B). Additionally, CD107a-positive NKdim cells from moderate and severe COVID-19 patients demonstrated greater activation levels than those obtained from healthy controls (Supplementary Figure S4A).

Notably, the cytotoxic activity by NKdim cells was significantly increased in deceased patients compared to those who survived COVID-19 disease in the ICU (Figure 8E). In contrast, NKbright cells derived from deceased patients exhibited lower activation levels (CD69 expression) than cells from patients who survived (Figure 8F). This discrepancy can be attributed to the decreased CD69 expression in CD107a negative cells of the NKbright subset compared to their CD107a positive counterparts (Figure 8G). Moreover, a substantial, albeit not statistically significant, decrease in activity was observed in CD107a negative NKbright cells of deceased patients compared to the survivors (Figure 8G).

**Figure 9:**
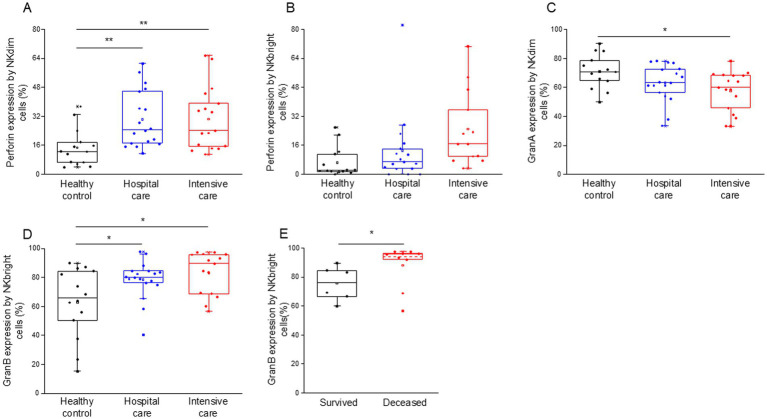
Perforin, granzyme B and granzyme A content of the different NK cell subpopulations in patients with moderate or severe COVID-19 and healthy controls. Intracellular perforin expression by NKdim **(A)** and NKbright **(B)** subpopulations in patients with moderate or severe COVID-19 infection and healthy controls. Granzyme A expression by NKdim **(C)** and granzyme B expression by NKbright **(D)** subpopulations in patients with moderate or severe COVID-19 infection and healthy controls. Granzyme B expression by NKbright subpopulation in survived and deceased patients **(E)**. The solid bars represent medians, the boxes indicate the interquartile ranges, and the lines show the most extreme observations. Differences were considered statistically significant for *p*-values ≤0.05. **p < 0.01, *p < 0.05.

Flow cytometric analysis further revealed a notable increase in intracellular perforin expression in both NK cell subpopulations, with NKdim cells showing increased expression in both IDU and ICU patients (Figure 9A). In contrast, NKbright cells exhibited no difference in the expression of perforin in ICU patients compared to healthy individuals (Figure 9B). Patients with severe disease displayed a significant decrease in granzyme A expression, specifically in NKdim cells (Figure 9C). Conversely, NKbright cells in both patient groups exhibited significantly elevated levels of granzyme B expression (Figure 9D), with a higher expression observed in deceased patients (Figure 9E).

A significant positive relationship was detected between the intracellular perforin content of NKdim cells and the level of soluble perforin in patients who survived COVID-19 infection (Supplementary Figure S5).

As previously demonstrated, NKT cells exhibit a cytotoxic profile characterized by the expression of cytolytic proteins such as granzyme A, B and perforin (Barakonyi et al., 2014). Consequently, we investigated the functional properties of NKT cells in patients and healthy controls.

**Figure 10:**
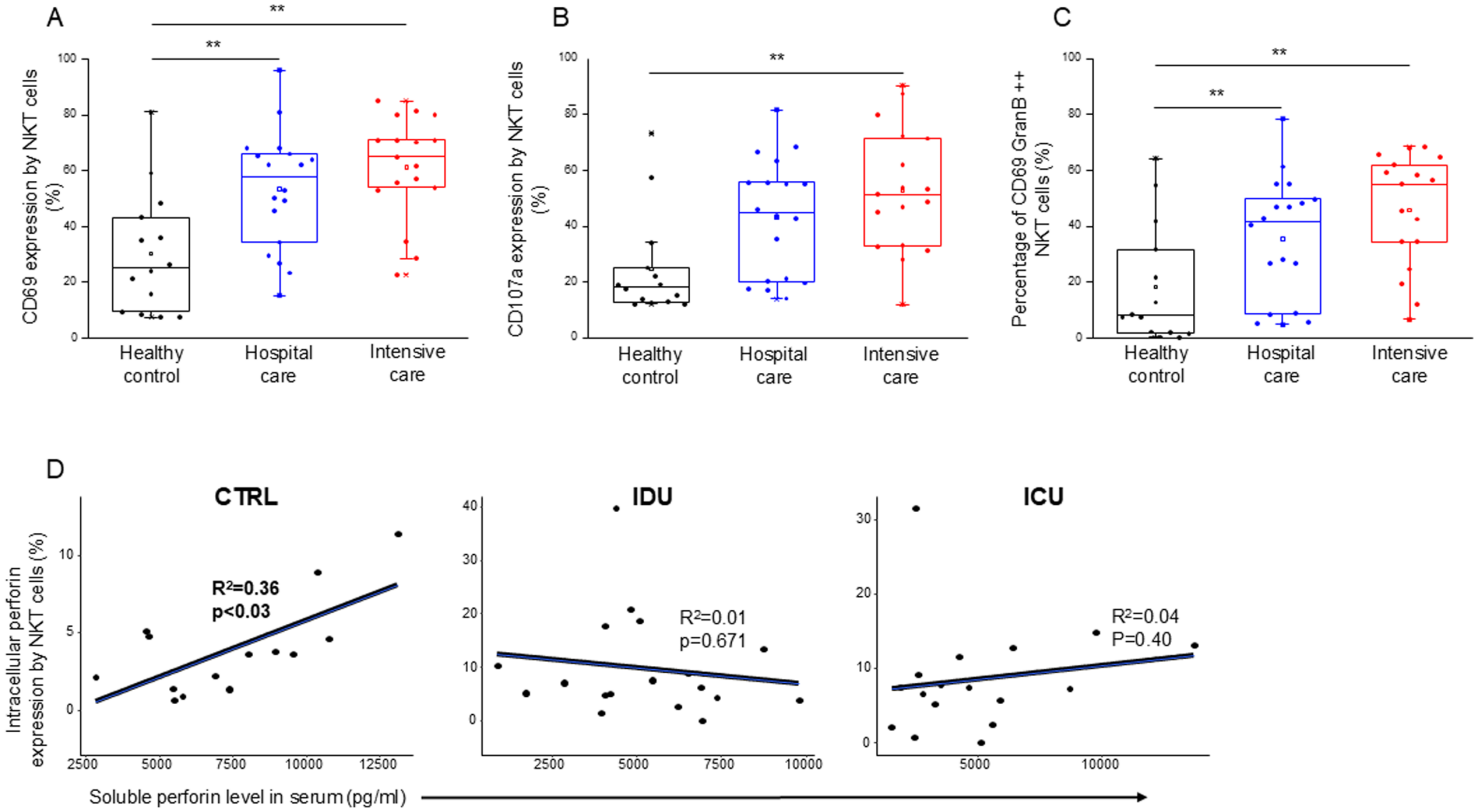
Cytotoxic activity of the NKT cell subpopulation in patients with moderate or severe COVID-19 and healthy controls. CD69 **(A)** and CD107a **(B)** expression by NKT cells in patients with moderate or severe COVID-19 and healthy controls. The percentage of the CD69 and granzyme B double-positive NKT cells in patients with moderate or severe COVID-19 infection and healthy controls **(C)**. Linear regression analyses between the soluble level of perforin and the intracellular level of perforin by NKT cells in patients with moderate or severe COVID-19 infection and healthy controls **(D)**. *p* values and coefficients of determination (R2) were calculated in R. **p < 0.01.

NKT cells from patients with moderate and severe disease showed a significantly more activated phenotype, as indicated by the CD69 expression, than healthy controls (Figure 10A). As characterized by CD107a expression, enhanced cytotoxicity was only observed in NKT cells from patients with severe disease (Figure 10B). A positive relation between CD69 and CD107a expression was revealed as cytotoxic cells positive for CD107a in all investigated groups (IDU, ICU and healthy group) displayed greater activation than their CD107a negative counterparts (Supplementary Figure S6).

Investigating NKT cells, there was no detectable difference during the analysis of any parameters between IDU and ICU patients. Similarly to patients with severe disease, NKT cells from moderate patients exhibited significantly higher CD69 expression and a significantly elevated frequency of CD69/Granzyme B double-positive NKT cells compared to healthy controls (Figure 10C). A significant positive relationship was detected between the intracellular perforin content of NKT cells and the level of soluble perforin in the control group (Figure 10D).

## Discussion

COVID-19 disease, the most significant global health concern in recent years, has led to severe and sometimes irreparable damage to human health. Several studies have compared patient parameters and profiles based on COVID-19 disease severity. However, no investigation has explored the differences in immune profiles between moderate and severe patients undergoing a cytokine storm, the impact of immune checkpoint pathways on hospitalized COVID-19 patients, and their association with mortality. Our study has a cross-sectional design; data collection occurred exclusively at the point of hospital admission for both moderate and severe cases of COVID-19 patients. Notably, no significant difference was observed in the time interval between the onset of symptoms and admission to either IDU or ICU. Nevertheless, it is conceivable that distinct immunological responses, though they did not influence the admission timing, may contribute to varying degrees of symptom severity, each necessitating the provision of professional inpatient medical care.

Examining immune checkpoint pathways in COVID-19 patients is crucial, as it sheds light on their presence and activity levels, contributing to disease severity and unfavorable clinical outcomes. To address this question, we directed our attention to IC ligands expressed by monocytes, as accumulating evidence suggests that monocytes play a pivotal role in orchestrating dysregulated immune responses (Merad and Martin, 2020b).

Although lymphopenia is not fully understood in COVID-19 (André et al., 2022; Signore et al., 2022), the decline in peripheral T cell numbers is among the most common findings in patients with severe disease (Al-Mterin et al., 2022). In some patients, lymphopenia has been reported to involve CD4+ T cells, CD8+ T cells, B cells and NK cells (Chen and John, 2020). In contrast, other data suggest that SARS-CoV-2 infection preferentially impacts CD8+ T cells (Mazzoni et al., 2020). It remains unclear why the lymphopenia is T cell-biased and perhaps specifically CD8+ T cell-biased, the presence of SARS-CoV-2 specific CD4+ and CD8+ T cells have been associated with milder disease (Rovito et al., 2022), consistent with the observation that patients with moderate disease displayed a T cell count similar to that of healthy controls. Noteworthy findings emerged from comparing the two patient populations.

A recent study showed that CD8+ T cells and NK cells in severe COVID-19 cases were reduced in numbers but exhibited hyperactivity (Jiang et al., 2020). Consistent with these reports, we observed a significant decrease in CD8+ T cell frequency in our severe patient group compared to moderate patients and healthy controls aligning with the critical role of T cell response in controlling viral infection. Some researchers have hypothesized T-cell exhaustion based on the overexpression of inhibitory immune checkpoint receptors (e.g., TIM-3 and PD-1) by CD8+ T cells (Gambichler et al., 2024; Zheng et al., 2020; Cao, 2020). However, the upregulation of these receptors may also indicate a hyperactivation signature in CD8+ T cells, characterized by elevated expression of NK cell-related markers and increased cytotoxicity (Chen and John, 2020; Mathew et al., 2020).

Non-classical monocytes were significantly reduced in both patient groups, suggesting a potential marker for severe COVID-19 (Silvin et al., 2020). A recent study conducted on bronchoscopy samples from COVID-19 patients admitted to the ICU has also found an enrichment of non-classical monocytes in the lungs (Sánchez-Cerrillo et al., 2020). Consistent with the literature reports (Vanderbeke et al., 2021; Rutkowska et al., 2022; Wen et al., 2020; Lee et al., 2020), we observed an increase in the frequency of classical subpopulations in all patients, reaching statistical significance only in patients with moderate disease compared to healthy controls. The upregulation of immune checkpoint ligand expressions was observed in monocytes across moderate and severe cases, suggesting a strive to mitigate the hyperreactivity. This phenomenon is noteworthy, as monocytes are principal regulators of various immune cell populations.

The retained PD-1 receptor expression by CD8+ T cells and the upregulated ligand expression by monocytes in the peripheral blood may represent an overall inhibition through the PD-1/PD-L1 pathway. Notably, we found that PD-L1 may have a prognostic value in severe patients, supported by a genomic analysis conducted by Fredericks et al. (2022) in infected patients.

Elevated soluble PD-1 levels in severe patients align with Cortés-Vieyra et al. (2024), though discrepancies with Beserra et al. (2022) highlight variability in patient cohorts. This discrepancy highlights the complexity of immune checkpoint regulation in COVID-19 and suggests that the dynamics of soluble versus cell-bound forms of these molecules may vary depending on patient cohorts and disease conditions.

To our knowledge, no prior studies in the literature evaluate CD112 or CD155 expression by monocytes in COVID-19 patients. The altered expression of immune checkpoint ligands (CD112 and CD155) by intermediate and non-classical monocytes may have implications for immune responses during COVID-19 infection, as these ligands can interact with the co-inhibitory receptor TIGIT and the activating receptor CD226. TIGIT not only binds with a much higher affinity to CD155 than to CD112, but this binding is much stronger compared to the one found regarding CD226. A major difference was found in TIGIT expression by CD8+ T and NK cells between IDU and ICU patients, with moderate cases having significantly lower values than healthy ones. Lower levels of immune checkpoint receptors would help activate immune cells, as it could be observed in moderate cases.

In severe cases, the expression of TIGIT reached a level comparable to that observed in healthy individuals, yet concomitant hyperactivation of CD8+ T cells and NK cells was detected. In the case of NKT cells, PD-1 expression was significantly elevated among ICU patients, notwithstanding the concurrent increased activation of NKT cells. Severe cases suggest resistance to immune checkpoint inhibition, exacerbating the immune response. To investigate this further, it is advisable to conduct repeated measurements at the point of discharge for initially moderate patients and in later stages of severe disease in ICU patients.

Since viral infections can affect the cytolytic granule production and cytotoxicity of CD8+ T cells, we evaluated the intracellular expression of perforin, granzyme A and granzyme B, along with the surface expression of CD107a molecule. The increase in intracellular perforin levels is observed in almost every cell population during COVID-19 infection (IDU + ICU), along with a simultaneous decrease in serum levels, indicating that they release lower perforin. An increased expression of CD69 was detected by the perforin+ CD8+ T cells in the deceased group. Furthermore, intracellular perforin content and serum perforin levels show a significant positive correlation. The explanation could be that CD8+ T cells are more activated in deceased individuals and release perforin (proportionally to their production) due to activation. This contributes to the exaggerated immune response in the deceased, which is also supported by the increased (albeit only a trend) CD107a expression by CD8+ T cells.

A significant positive relationship was detected within the control group between the intracellular perforin content of NKT cells and the level of soluble perforin, suggesting a potential equilibrium state in the control group, which could be associated with a mild Th1-oriented immune response characteristic of a healthy physiological condition. In the context of COVID-19 infection, a reduction in soluble perforin levels was concomitant with an elevation in PD-1 expression by NKT cells. The expression of cell surface molecules linked to cytotoxicity, such as CD107a and CD69, exhibited an increase in NKT cells despite the absence of alteration in the intracellular content of cytotoxic molecules, even though an increase was observed in other cell populations. This phenomenon can likely be attributed to the PD-1 inhibitory receptor. Furthermore, the positive correlation observed in the control group is notably absent during COVID-19 infection primarily due to the reduced perforin production, resulting in an insufficient release of perforin into the circulation.

Elevated inhibitory immune checkpoint receptor expression has often been associated with functional T cell exhaustion (Zheng et al., 2020; Cao, 2020; Zinselmeyer et al., 2013). However, findings from this study indicate that peripheral CD8+ T cells in severe COVID-19 patients exhibit characteristics of hyperactivation rather than a fully exhausted phenotype. The elevated immune checkpoint expression observed in severe COVID-19 appears to reflect a spectrum of immune dysfunction, encompassing both hyperactivation and features of exhaustion. This dual phenomenon likely contributes to the impaired viral clearance and the exacerbation of inflammation characteristic of severe disease. These findings are supported by the observed increase in cytotoxic activity and checkpoint marker expression, highlighting the complex interplay between immune activation and regulatory dysfunction in severe COVID-19.

We must note that the present study has limitations. First, the relatively small sample size limits the statistical power and prevents further desired analyses. Second, our experiments focused on peripheral blood cells, potentially overlooking organ-specific immune processes in specific microenvironments. Third, despite investigating a wide range of immune cell types, we were unable to examine other crucial cell types involved in innate and adaptive immune response, such as dendritic cells, granulocytes and B cells. Fourth, analyzing the co-expression of immune checkpoint receptors on immune cells can be very informative. Still, we were unable to run the detection of all the receptors in a single panel. Fifth, longitudinal studies assessing immune cell responses over time were not conducted, which could provide valuable insights into immune changes. Sixth, our cohort’s limited number of deceased patients restricts a meaningful correlation between disease outcomes and immunological responses. Lastly, the mean age of ICU patients was significantly higher than that of the enrolled IDU patients and healthy individuals, which could be attributed to the generally observed more severe nature of the disease in older age.

The data presented here provides novel insights into the immune response regulation in COVID-19 patients with cytokine storms, highlighting potential differences between moderate or severe clinical states. It is plausible that disease severity may vary among patients depending on their immune response spectrum. However, the relationship between immune response intensity in peripheral blood, respiratory tract, and other SARS-CoV-2-infected organs remains unclear. These findings highlight distinct immune checkpoint pathways and activation signatures in non-surviving patients compared to survivors with COVID-19, underscoring the association between high innate cell activation, increased cytotoxicity of NKdim cells, and fatal outcomes.

The observed upregulation of PD-1 and PD-L1, along with increased TIGIT and CD112/CD155 expression in severe cases, suggests that these markers may serve as potential biomarkers for identifying high-risk COVID-19 patients with worse prognosis and the need for more intensive clinical monitoring. Such markers could aid clinicians in stratifying patients based on disease severity and predicting outcomes, thereby guiding therapeutic decisions, particularly regarding immunomodulatory treatments.

This paper summarizes our recent findings on the potential involvement of immune checkpoint molecules in COVID-19 pathogenesis, focusing on disease severity. Our observations suggest a negative role for different immune cells and their subsets in severe COVID-19 infection.

## Conclusion

This research unveils the intricate regulation of immune responses in COVID-19 and underscores the pivotal role of immune checkpoint pathways and activation signatures in determining disease severity. These findings have far-reaching implications for our understanding of COVID-19 pathogenesis and the development of potential therapeutic interventions.

## Data Availability

The raw data supporting the conclusions of this article will be made available by the authors, without undue reservation.
