# Supplementary material for: Can there be calm during a cytokine storm? Immune checkpoint pathways affecting the severity of COVID-19 disease
Source: Front Microbiol. 2024 Dec 23;15:1508423. doi: 10.3389/fmicb.2024.1508423 (PMC11700970; doi:10.3389/fmicb.2024.1508423)
Supplement: Supplementary file 1 [file Table_1.docx]

**Table 1. Demographic characteristics and comorbidities of patients on admission**

| **Variables** | **Healthy controls**  **(n=14)** | **IDU patients**  **(n=18)** | **ICU patients**  **(n=17)** | **p-value** |
| --- | --- | --- | --- | --- |
| Age, mean, years (range) | 44  (16-73) | 52.2  (19-66) | 66  (46-86) | **<0.05 HC vs. ICU**  **<0.05 IDU vs. ICU** |
| Females/males | 6 / 8 | 6 / 12 | 6 / 11 | NS |
| Obesity (BMI, median) | NA | 27.78  (19.11, 45.2) | 29.4  (21.48, 71.78) | NS |
| **Comorbidities** |  |  |  |  |
| Hypertension (n, %) | 0 | 6 (33.3%) | 14 (82.4%) | **<0.01 ICU vs. IDU** |
| Heart disease (n, %) | 0 | 0 | 3 (17.6%) | NS |
| Chronic lung disease (n, %) | 0 | 1 (5.5%) | 0 | NS |
| Chronic kidney disease (n, %) | 0 | 0 | 2 (11.7%) | NS |
| Diabetes (n, %) | 0 | 2 (11.1%) | 6 (35.3%) | NS |
| Days elapsed between the onset of symptoms and admission | - | 6.7 ± 3.6 | 5.4 ± 4.1 | NS |

Data are presented as mean ± standard deviation, median (minimum, maximum) or numbers and percentages. Differences were considered significant when the P value was equal to or less than 0.05. Comparison of IDU vs. ICU patients on the day of admission vs. healthy controls. ICU: Intensive care unit, IDU: infectious disease unit, HC: healthy control, BMI: body mass index; NS: not significant; NA: data not available. Significant results are presented in bold.

**Table 2. Laboratory and clinical characteristics of ICU and IDU patients**

| **Laboratory parameters** | **IDU patients (n=18)** | **ICU patients (n=17)** | **p-value** |
| --- | --- | --- | --- |
| Lymphocyte count (cells/mm^3^) | 0.8 ± 0.17 | 0.66 ± 0.21 | **<0.01** |
| LDH (U/L) | 431 ± 232 | 926 ± 476 | **<0.001** |
| D-dimer (ng/ml) | 1027 (228, 24582) | 1597 (681, 329800) | NS |
| Ferritin (ng/ml) | 1035 (42, 6155) | 1117 (32, 9667) | NS |
| CRP (mg/dl) | 9.6 ± 6.7 | 12.8 ± 7.7 | NS |
| IL-6 (pg/ml) | 62 (7, 143) | 61 (31, 924) | NS |
| **Clinical parameters** |  |  |  |
| Days of hospitalization | 8 ± 5 | 15 ± 12 | **<0.05** |
| Mortality (n, %) | 0 | 11 (64.7%) |  |
| SOFA | 2 (0.4) | 3 (0, 9) | **<0.05** |
| SAPS | - | 33 (13, 71) |  |
| CT score | 11 ± 4.8 | 15 ± 6.8 | **<0.05** |
| PaO_2_/FiO_2_ on admission | 194 (32, 410) | 67 (43, 388) | **<0.05** |
| Mechanical ventilation (days) | - | 4 (0, 19) |  |
| Kidney replacement therapy (n, %) | - | 3 (17%) |  |
| Vasopressor therapy (n, %) | - | 13 (76%) |  |

Data are presented as mean ± standard deviation or median (minimum, maximum). Differences were considered significant when the P value was equal to or less than 0.05. ICU: Intensive care unit, IDU: infectious disease unit HC: healthy control, LDH: lactate dehydrogenase; CRP: C-reactive protein; IL-6: interleukin-6; SOFA: Sequential organ failure assessment score; SAPS, simplified acute physiology score; CT score: computed tomography score; NS: not significant. Significant results are presented in bold.

**Table 3.:** Phenotype analysis of peripheral blood mononuclear cells

|  | **Gate** | **Healthy controls**  **(n=14)** | **IDU patients**  **(n=18)** | **ICU patients**  **(n=17)** | **p-value** |
| --- | --- | --- | --- | --- | --- |
| **CD3+ T cells** | lymphogate | 57.10±10.63 | 56.13±9.93 | 50.38±11.97 | NS |
| **CD4+ T cells** | lymphogate | 30.20±13.88 | 29.50±10.93 | 32.14±13.64 | NS |
| **CD4+ T cells in CD3+ T cells** | lymphogate | 50.70±16.61 | 52.66±16.34 | 62.21±14.75 | NS |
| **CD8+ T cells** | lymphogate | 20.82±4.60 | 20.57±8.52 | 14.88±7.41 | **<0.05 HC vs. ICU**  **<0.05 IDU vs. ICU** |
| **CD8+ T cells in CD3+ T cells** | lymphogate | 37.86±11.31 | 36.80±14.01 | 30.81±15.39 | NS |
| **NK cells** | lymphogate | 17.86±8.17 | 19.99±7.97 | 21.87±12.54 | NS |
| **NK^dim^ cells** | lymphogate | 17.30±8.10 | 19.15±7.75 | 21.24±12.38 | NS |
| **NK^bright^ cells** | lymphogate | 0.58±0.33 | 0.86±0.65 | 0.66±1.01 | NS |
| **NKT-like cells** | lymphogate | 7.98±6.69 | 7.62±7.65 | 5.47±4.19 | NS |
| **Classical monocytes** | monocyte | 94.98±2.65 | 98.07±1.27 | 96.85±0.92 | **<0.05 HC vs. IDU** |
| **Intermediate monocytes** | monocyte | 1.31±0.94 | 1.25±0.93 | 1.95±4.35 | NS |
| **Non-classical**  **monocytes** | monocyte | 3.61±1.95 | 0.37±0.36 | 0.74±0.79 | **<0.01 HC vs. IDU**  **<0.01 HC vs. ICU** |

The results were expressed in percentages of cells as the mean value ± standard deviation of the mean (SD). Differences were considered significant when the P value was equal to or less than 0.05. ICU: Intensive care unit, IDU: infectious disease unit HC: healthy control, NS: not significant. Significant results are presented in bold.

**Table 4.:** Soluble level (pg/ml) of immune checkpoint and cytotoxic molecules

| **pg/ml** | **Healthy controls**  **(n=14)** | **IDU patients**  **(n=18)** | **ICU patients**  **(n=17)** | **p-value** |
| --- | --- | --- | --- | --- |
| **PD-1** | 177.40±48.93 | 202.86±108.66 | 315.27±235.33 | **<0.05 HC vs. ICU** |
| **PD-L1** | 18.01±11.20 | 21.28±17.17 | 13.56±5.72 | NS |
| **CD226** | 5241.56±6446.11 | 3434.97±3102.36 | 2246.97±1981.54 | NS |
| **CD155** | 25306.33±13593.00 | 36508.44±17912.19 | 46459.86±17912.19 | **<0.01 HC vs. ICU** |
| **CD112** | 644.15±195.62 | 1070.89±324.33 | 1854.79±675.19 | **<0.01 HC vs. ICU**  **<0.01 IDU vs. ICU**  **<0.05 HC vs. IDU** |
| **Perforin** | 7418.56±2862.88 | 5224.87±2288.21 | 4988.57±3214.66 | **<0.052 HC vs. ICU** |
| **Granzyme B** | 3.31±1.46 | 15.04±10.77 | 18.00±20.91 | **<0.06 HC vs. IDU**  **<0.05 HC vs. ICU** |

The results were expressed as the mean value ± SD of the mean. Differences were considered significant when the P value was equal to or less than 0.05. ICU: Intensive care unit, IDU: HC: healthy control, NS: not significant. Significant results are presented in bold.
