# Supplementary material for: Can there be calm during a cytokine storm? Immune checkpoint pathways affecting the severity of COVID-19 disease
Source: Front Microbiol. 2024 Dec 23;15:1508423. doi: 10.3389/fmicb.2024.1508423 (PMC11700970; doi:10.3389/fmicb.2024.1508423)
Supplement: Supplementary file 2 [file Table_2.docx]

**Supplementary table 1.** Fluorochrome conjugated monoclonal antibodies used in the study.

| **Antigen** | **Format** | **Clone** | **Isotype** | **Company** | **CAT** |
| --- | --- | --- | --- | --- | --- |
| **CD3** | BV510 | UCHT1 | Mouse BALB/c IgGκ | BD Biosciences | 563109 |
| **CD4** | FITC | RPA-T4 | Mouse IgG1, κ | BD Biosciences | 555346 |
| **CD8** | APC-H7 | SK1 | Mouse BALB/c IgG1, κ | BD Biosciences | 560179 |
| **CD14** | FITC | M5E2 | Mouse IgG2a, κ | BD Biosciences | 555397 |
| **CD16** | PerCp Cy5.5 | 3G8 | Mouse CDF1 IgG1, κ | BD Biosciences | 560717 |
| **CD56** | APC | B159 | Mouse IgG1, κ | BD Biosciences | 555518 |
| **CD69** | BV421 | FN50 | Mouse IgG1, κ | BD Biosciences | 562884 |
| **CD107a** | FITC | H4A3 | Mouse BALB/c IgG1, κ | BD Biosciences | 555800 |
| **CD112** | PE | R2.525 | Mouse IgG1, κ | BD Biosciences | 551057 |
| **CD155** | APC | SKII.4 | Mouse IgG1, κ | Biolegend | 337618 |
| **CD161** | APC | 191B8 | Mouse IgG2a, κ | Miltenyi Biotec | 130113590 |
| **CD169** | PE-Cy7 | 7-239 | Mouse IgG1, κ | Biolegend | 346014 |
| **CD226** | BV421 | DX11 | Mouse BALB/c IgG1, κ | BD Biosciences | 742493 |
| **Granzyme A** | PerCp Cy5.5 | CB9 | Mouse IgG1, κ | Biolegend | 507216 |
| **Granzyme B** | PE | GB11 | Mouse BALB/c IgG1, κ | BD Biosciences | 561142 |
| **HLA-DR** | APC-H7 | G46-6 | Mouse IgG2a, κ | BD Biosciences | 561358 |
| **PD-1** | PerCp Cy5.5 | EH12.1 | Mouse IgG1, κ | BD Biosciences | 561273 |
| **PD-L1** | BV421 | MIH1 | Mouse BALB/c IgG1, κ | BD Biosciences | 563738 |
| **Perforin** | PE-Cy7 | dG9 | Mouse IgG2b, κ | Biolegend | 308126 |
| **TIGIT** | PE | A1553G | Mouse IgG2a,  | Biolegend | 372704 |
| **Va72** | FITC | 3C10 | Mouse IgG1, κ | Biolegend | 351704 |

**Supplementary table 2.** PD-1 expression by peripheral blood mononuclear cell phenotype characteristics in patients with moderate or severe COVID-19 infection and healthy controls

| **PD-1 expression (%)** | | | | | |  |
| --- | --- | --- | --- | --- | --- | --- |
|  | **Healthy controls**  **(n=14)** | **IDU patients**  **(n=17)** | **ICU patients**  **(n=16)** | **Survived**  **ICU patients**  **(n=6)** | **Deceased**  **ICU patients**  **(n=10)** | **p-value** |
| **CD3+ T** | 17.75±5.81 | 17.75±7.04 | 22.96±10.79 | 28.06±12.32 | 19.91±9.06 | NS |
| **CD4+ T** | 14.11±6.48 | 17.14±8.71 | 21.16±11.63 | 27.00±14.75 | 17.66±8.26 | NS |
| **CD8+ T** | 22.54±7.21 | 20.81±9.89 | 26.30±11.98 | 29.77±13.69 | 24.22±11.05 | NS |
| **NK** | 5.39±5.55 | 7.43±5.91 | 8.01±6.23 | 7.79±8.14 | 8.15±5.27 | NS |
| **NKdim** | 5.35±5.50 | 7.39±5.89 | 8.03±6.25 | 7.82±8.16 | 8.16±5.31 | NS |
| **NKbright** | 6.58±6.29 | 7.54±6.43 | 7.52±7.94 | 6.77±7.56 | 8.08±8.68 | NS |
| **NKT** | 18.59±8.48 | 26.95±14.34 | 34.94±19.65 | 34.16±14.67 | 35.33±22.46 | **<0.05 HC vs. ICU** |

The results were expressed as the mean value ± standard deviation of the mean (SD). Differences were considered significant when the value of P was equal to or less than 0.05. ICU: Intensive care unit, IDU: infectious disease unit, HC: healthy control, NS: not significant. Significant results are presented in bold.

**Supplementary table 3.** PD-L1 expression by peripheral blood mononuclear cell phenotype characteristics in patients with moderate or severe COVID-19 infection and healthy controls

| **PD-L1 expression (%)** | | | | | |  |
| --- | --- | --- | --- | --- | --- | --- |
|  | **Healthy controls**  **(n=14)** | **IDU patients**  **(n=18)** | **ICU patients**  **(n=17)** | **Survived**  **ICU patients**  **(n=6)** | **Deceased**  **ICU patients**  **(n=11)** | **p-value** |
| **Classical**  **monocytes** | 4.82±9.89 | 27.62±27.24 | 31.42±16.26 | 20.48±9.63 | 37.39±16.30 | **<0.01 HC vs. IDU**  **<0.01 HC vs. ICU**  **<0.01 survived vs. deceased** |
| **Intermediate**  **monocytes** | 10.74±13.38 | 53.88±26.61 | 57.75±21.86 | 47.75±16.40 | 63.21±23.18 | **<0.01 HC vs. IDU**  **<0.01 HC vs. ICU** |
| **Non-classical**  **monocytes** | 5.76±7.83 | 35.52±27.23 | 32.55±16.98 | 18.52±10.59 | 40.97±14.43 | **<0.01 HC vs. IDU**  **<0.01 survived vs. deceased** |

The results were expressed as the mean value ± standard deviation of the mean (SD). Differences were considered significant when the P value was equal to or less than 0.05. ICU: Intensive care unit, IDU: infectious disease unit, HC: healthy control. Significant results are presented in bold.

**Supplementary table 4.** CD226 expression by peripheral blood mononuclear cell phenotype characteristics in patients with moderate or severe COVID-19 infection and healthy controls

| **CD226 expression (%)** | | | | | |  |
| --- | --- | --- | --- | --- | --- | --- |
|  | **Healthy controls**  **(n=14)** | **IDU patients**  **(n=17)** | **ICU patients**  **(n=16)** | **Survived**  **ICU patients**  **(n=6)** | **Deceased**  **ICU patients**  **(n=10)** | **p-value** |
| **CD3+ T** | 66.69±13.22 | 55.84±15.08 | 58.38±11.62 | 60.38±9.04 | 57.30±13.09 | NS |
| **CD4+ T** | 63.41±13.44 | 54.92±17.15 | 58.28±14.40 | 58.87±13.53 | 57.96±15.49 | NS |
| **CD8+ T** | 67.19±16.53 | 58.50±15.39 | 62.74±11.55 | 62.87±12.42 | 62.67±11.68 | NS |
| **NK** | 74.57±12.49 | 65.90±14.36 | 63.50±10.73 | 67.47±11.59 | 61.33±10.12 | NS |
| **NK^dim^** | 74.35±12.56 | 65.20±13.64 | 63.15±10.81 | 67.26±11.45 | 60.90±10.28 | NS |
| **NK^bright^** | 81.73±10.97 | 73.73±17.00 | 73.34±13.16 | 75.04±14.85 | 72.21±12.71 | NS |
| **NKT** | 85.52±20.08 | 85.31±11.88 | 77.17±16.10 | 81.52±17.55 | 74.80±15.59 | NS |

The results were expressed as the mean value ± standard deviation of the mean (SD). Differences were considered significant when the P value was equal to or less than 0.05. ICU: Intensive care unit, IDU: infectious disease unit, HC: healthy control, NS: not significant.

**Supplementary table 5.** TIGIT expression by peripheral blood mononuclear cell phenotype characteristics in patients with moderate or severe COVID-19 infection and healthy controls

| **TIGIT expression (%)** | | | | | |  |
| --- | --- | --- | --- | --- | --- | --- |
|  | **Healthy controls**  **(n=14)** | **IDU patients**  **(n=17)** | **ICU patients**  **(n=16)** | **Survived**  **ICU patients**  **(n=6)** | **Deceased**  **ICU patients**  **(n=10)** | **p-value** |
| **CD3+ T** | 22.52±8.93 | 12.61±6.45 | 22.70±11.35 | 24.71±8.81 | 21.50±12.93 | **<0.05 HC vs. IDU**  **<0.01 IDU vs. ICU** |
| **CD4+ T** | 13.10±5.85 | 8.56±5.70 | 12.23±6.64 | 13.17±7.21 | 11.66±6.61 | NS |
| **CD8+ T** | 36.27±15.59 | 20.21±8.87 | 39.53±14.76 | 41.99±13.52 | 38.06±15.97 | **<0.05 HC vs. IDU**  **<0.01 IDU vs. ICU** |
| **NK** | 40.09±10.05 | 25.92±14.09 | 30.35±11.52 | 32.38±10.95 | 29.14±12.26 | **<0.01 HC vs. IDU** |
| **NKdim** | 40.87±10.13 | 26.63±14.73 | 30.66±12.00 | 32.45±10.90 | 29.60±13.06 | **<0.01 HC vs. IDU** |
| **NKbright** | 20.93±13.27 | 13.45±11.20 | 20.87±16.69 | 26.56±21.10 | 17.45±13.51 | NS |
| **NKT** | 38.58±25.03 | 28.60±16.25 | 45.27±20.24 | 50.83±20.92 | 41.94±20.17 | NS |

The results were expressed as the mean value ± standard deviation of the mean (SD). Differences were considered significant when the P value was equal to or less than 0.05. ICU: Intensive care unit, IDU: infectious disease unit, HC: healthy control, NS: not significant. Significant results are presented in bold.

**Supplementary table 6.** CD112 expression by peripheral blood mononuclear cell phenotype characteristics in patients with moderate or severe COVID-19 infection and healthy controls

| **CD112 expression (%)** | | | | | |  |
| --- | --- | --- | --- | --- | --- | --- |
|  | **Healthy controls**  **(n=14)** | **IDU patients**  **(n=18)** | **ICU patients**  **(n=17)** | **Survived**  **ICU patients**  **(n=6)** | **Deceased**  **ICU patients**  **(n=11)** | **p-value** |
| **Classical**  **monocytes** | 48.12±17.93 | 52.40±16.33 | 56.23±12.69 | 52.59±6.00 | 58.22±15.08 | NS |
| **Intermediate**  **monocytes** | 61.26±15.64 | 67.48±16.66 | 77.42±13.25 | 74.49±7.38 | 79.02±15.68 | **<0.05 HC vs. ICU** |
| **Non-classical**  **monocytes** | 46.09±17.18 | 68.19±19.10 | 66.91±18.99 | 61.22±11.46 | 70.01±21.94 | NS |

The results were expressed as the mean value ± standard deviation of the mean (SD). Differences were considered significant when the P value was equal to or less than 0.05. ICU: Intensive care unit, IDU: infectious disease unit, HC: healthy control, NS: not significant. Significant results are presented in bold.

**Supplementary table 7.** CD155 expression by peripheral blood mononuclear cell phenotype characteristics in patients with moderate or severe COVID-19 infection and healthy controls

| **CD155 expression (%)** | | | | | |  |
| --- | --- | --- | --- | --- | --- | --- |
|  | **Healthy controls**  **(n=14)** | **IDU patients**  **(n=18)** | **ICU patients**  **(n=17)** | **Survived**  **ICU patients**  **(n=6)** | **Deceased**  **ICU patients**  **(n=11)** | **p-value** |
| **Classical**  **monocytes** | 55.64±20.32 | 47.25±16.35 | 57.54±14.42 | 53.19±8.24 | 59.91±16.77 | NS |
| **Intermediate**  **monocytes** | 37.17±13.47 | 49.52±19.53 | 68.90±14.54 | 60.94±14.60 | 73.24±13.16 | **<0.01 HC vs. ICU**  **<0.05 IDU vs. ICU** |
| **Non-classical**  **monocytes** | 27.96±10.20 | 26.19±15.23 | 38.05±19.37 | 30.95±24.61 | 41.92±15.84 | NS |

The results were expressed as the mean value ± standard deviation of the mean (SD). Differences were considered significant when the P value was equal to or less than 0.05. ICU: Intensive care unit, IDU: infectious disease unit, HC: healthy control, NS: not significant. Significant results are presented in bold.
