# Supplementary material for: Can there be calm during a cytokine storm? Immune checkpoint pathways affecting the severity of COVID-19 disease
Source: Front Microbiol. 2024 Dec 23;15:1508423. doi: 10.3389/fmicb.2024.1508423 (PMC11700970; doi:10.3389/fmicb.2024.1508423)
Supplement: Supplementary file 3 [file Table_3.docx]

**Supplementary figure 1. Flow cytometric data analyses to determine lymphocyte and monocyte subpopulations**

After a two-step doublet exclusion (A-B), the lymphocyte population was gated using FSC-A/SSC-A parameters (C). From the lymphogate, CD3⁺ T-, NK-, NKdim, NKbright-, NKT-, CD8⁺ T-, and CD4⁺ T cell subpopulations were detected (D-F). From the gated monocytes, the CD3⁺ T cell population was excluded (G). CD16 vs. HLA-DR dot plot: CD16⁺/HLA-DR- NK cells were excluded from the monocytes (H). CD14 vs. HLA-DR dot plot: HLA-DR high/CD14 low B cells were excluded from the monocytes (I). Using CD16 and CD14 markers, monocyte subpopulations were differentiated based on their characteristic “┐” shape (J).

**Supplementary figure 2. Regression analyses between the serum level of soluble PD-1 and the relative PD-1 expression by CD8+ T cells in patients with moderate or severe COVID-19 infection and healthy controls**

Linear regression analyses between the serum concentration of PD-1 and the relative expression of PD-1 by CD8+ T cells in patients with moderate or severe COVID-19 infection and in healthy controls. P values and coefficients of determination (R^2^) were calculated in R.

**Supplementary figure 3. Regression analyses between the serum level of soluble CD155 and relative CD155 expression by non-classical monocytes in patients with moderate or severe COVID-19 infection and healthy controls**

Linear regression analyses between the soluble level of CD155 and the relative expression of CD155 by non-classical monocytes in patients with moderate or severe COVID-19 infection and in healthy controls. P values and coefficients of determination (R^2^) were calculated in R.

**Supplementary figure 4. The activation level of NKdim and NKbright cells in patients with moderate or severe COVID-19 infection and healthy controls**

CD69 expression by CD107a positive and negative NKdim (A) and NKbright (B) cell subpopulations in patients with moderate or severe COVID-19 infection and healthy controls. The solid bars represent medians, the boxes indicate the interquartile ranges, and the lines show the most extreme observations. Differences were considered statistically significant for p-values ≤0.05. **p<0.01, *p<0.05

**Supplementary figure 5. Regression analyses between the serum level of soluble perforin and intracellular perforin content of NKdim cells in patients with moderate or severe COVID-19 infection and healthy controls**

Linear regression analyses between the soluble level of perforin and the intracellular level of perforin by NKdim cells in patients with moderate or severe COVID-19 infection and in healthy controls. P values and coefficients of determination (R^2^) were calculated in R.

**Supplementary figure 6. The activation level of NKT cells in patients with moderate or severe COVID-19 infection and healthy controls**

CD69 expression by CD107a positive and negative NKT cell subpopulation in patients with moderate or severe COVID-19 infection and healthy controls. The solid bars represent medians, the boxes indicate the interquartile ranges, and the lines show the most extreme observations. Differences were considered statistically significant for p-values ≤0.05. **p<0.01, *p<0.05
