# Supplementary material for: Can there be calm during a cytokine storm? Immune checkpoint pathways affecting the severity of COVID-19 disease
Source: Front Microbiol. 2024 Dec 23;15:1508423. doi: 10.3389/fmicb.2024.1508423 (PMC11700970; doi:10.3389/fmicb.2024.1508423)
Supplement: Supplementary file 4 [file Presentation_1.pptx]

## Slide 1
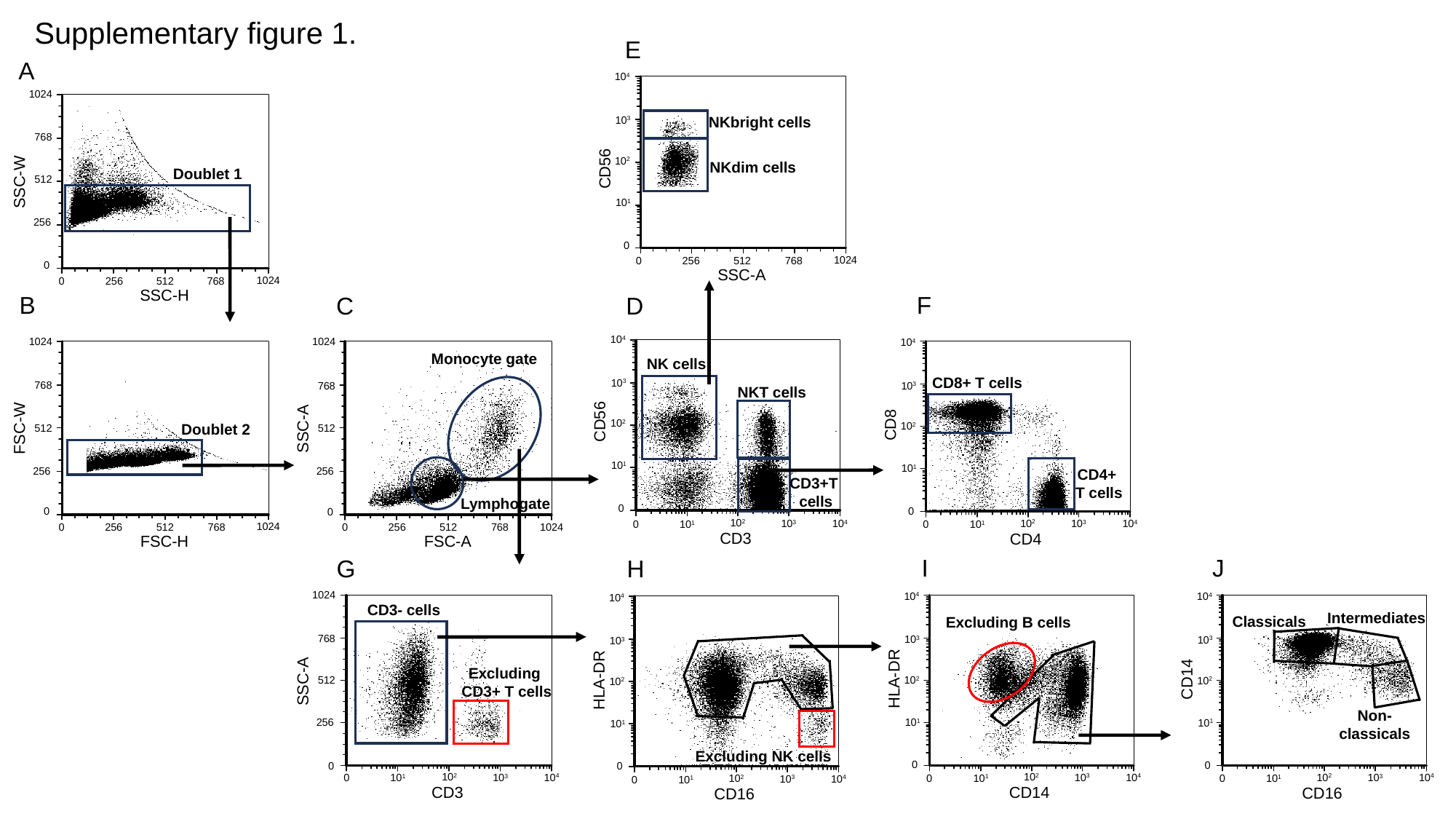

Supplementary figure 1.
E
A
104
1024
768
SSC-W
512
256
0
1024
512
0
256
768
SSC-H
NKbright cells
103
102
NKdim cells
CD56
Doublet 1
101
0
1024
512
0
256
768
SSC-A
F
B
D
C
104
103
CD56
102
101
0
102
104
103
101
0
CD3
1024
768
FSC-W
512
256
0
1024
512
0
256
768
FSC-H
1024
768
SSC-A
512
256
0
1024
512
0
256
768
FSC-A
104
103
102
101
0
102
104
103
101
0
CD4
Monocyte gate
NK cells
CD8+ T cells
NKT cells
CD8
Doublet 2
CD4+
T cells
CD3+T
cells
Lymphogate
I
J
H
G
1024
768
SSC-A
512
256
0
102
0
104
103
101
CD3
104
103
HLA-DR
102
101
0
102
104
103
101
0
CD14
104
103
CD14
102
101
0
102
104
103
101
0
CD16
104
103
HLA-DR
102
101
0
102
104
103
101
0
CD16
CD3- cells
Intermediates
Classicals
Excluding B cells
Excluding
CD3+ T cells
Non-classicals
Excluding NK cells

## Slide 2
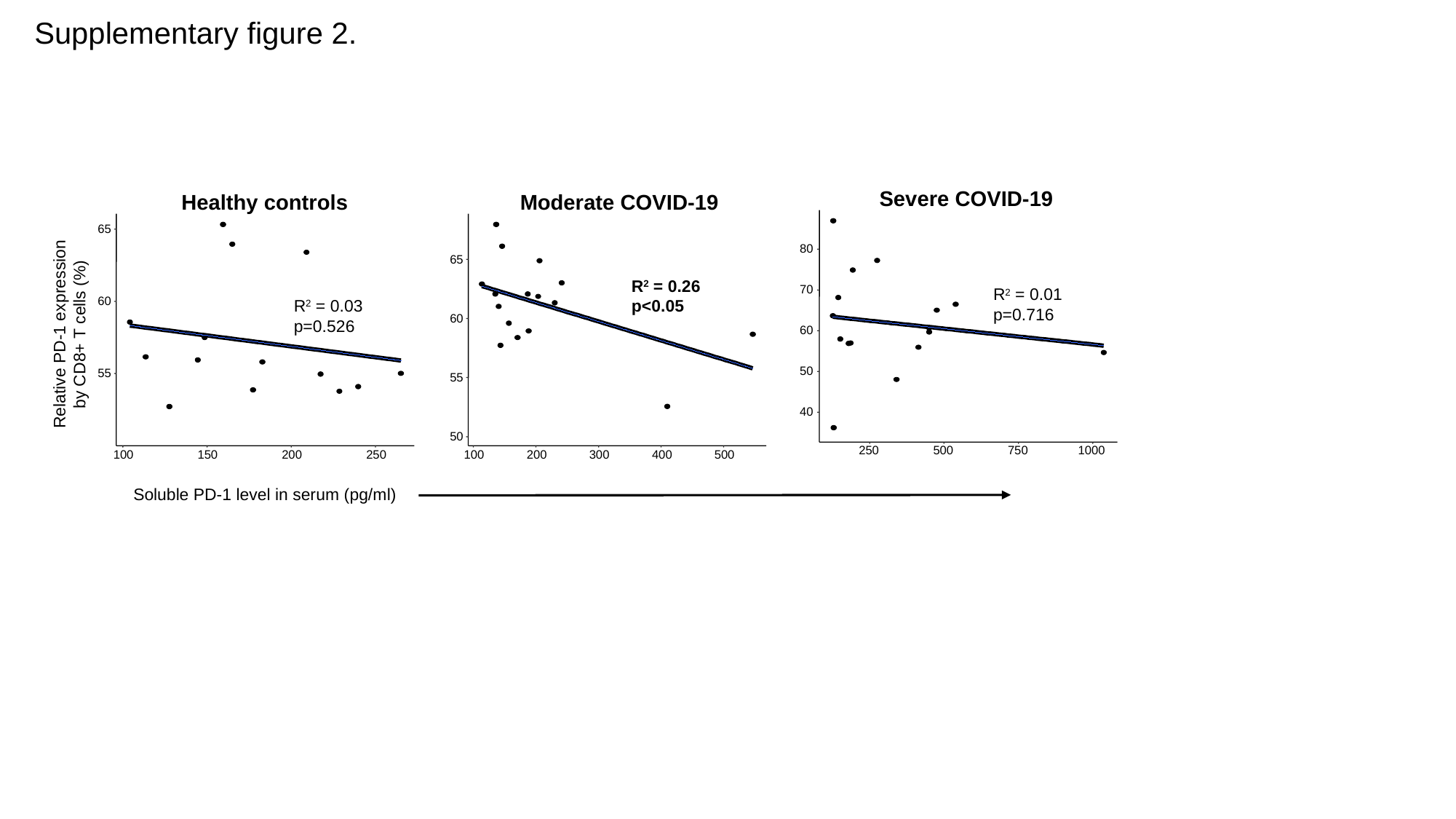

Supplementary figure 2.
Severe COVID-19
80
70
R2 = 0.01
p=0.716
60
50
40
750
1000
250
500
Healthy controls
65
60
R2 = 0.03
p=0.526
55
100
150
200
250
Moderate COVID-19
65
R2 = 0.26
p<0.05
60
55
50
100
200
300
400
500
Relative PD-1 expression by CD8+ T cells (%)
Soluble PD-1 level in serum (pg/ml)

## Slide 3
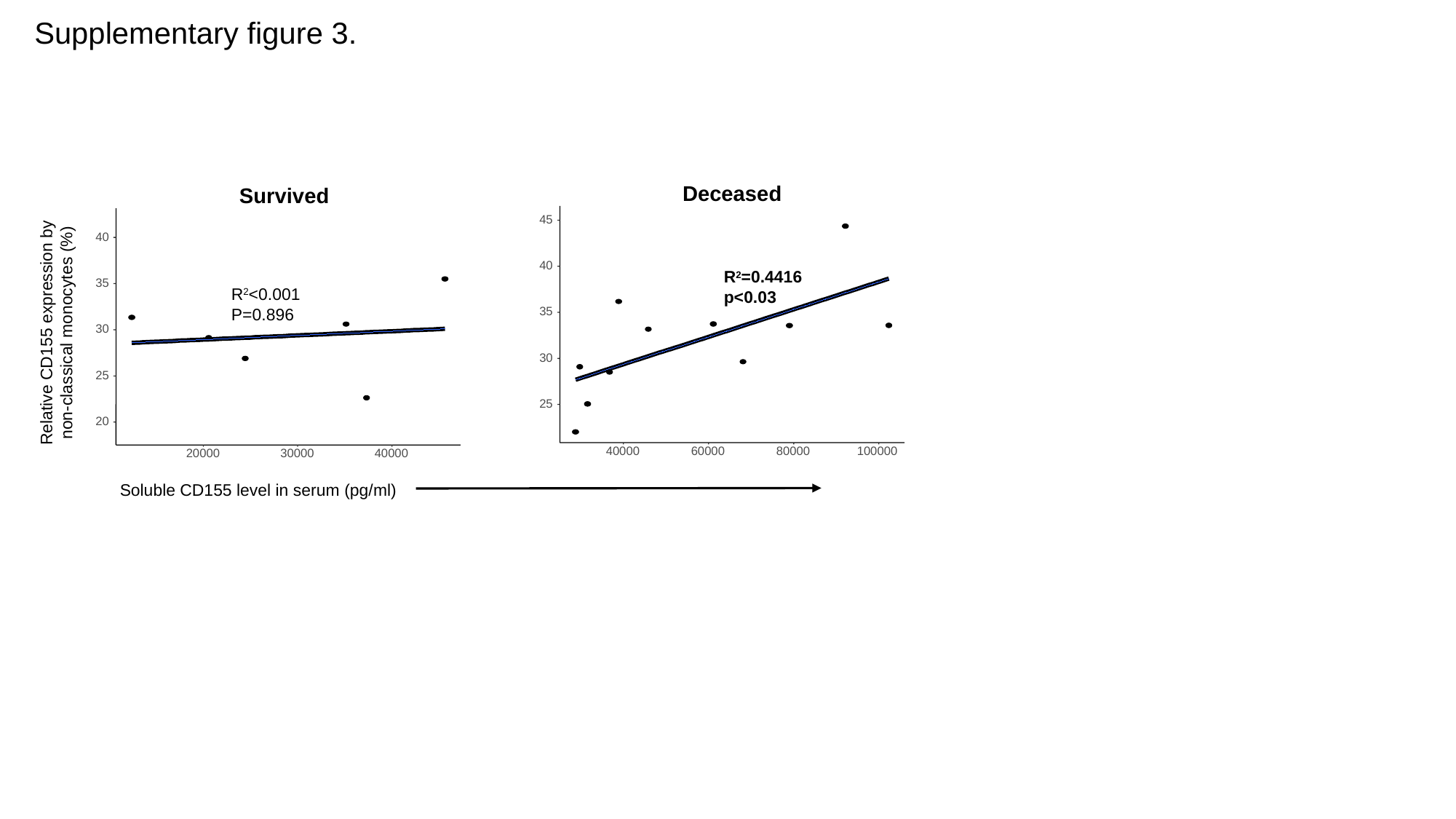

Supplementary figure 3.
Deceased
45
40
R2=0.4416
p<0.03
35
30
25
40000
60000
80000
100000
Survived
40
35
R2<0.001
P=0.896
30
25
20
20000
30000
40000
Relative CD155 expression by non-classical monocytes (%)
Soluble CD155 level in serum (pg/ml)

## Slide 4
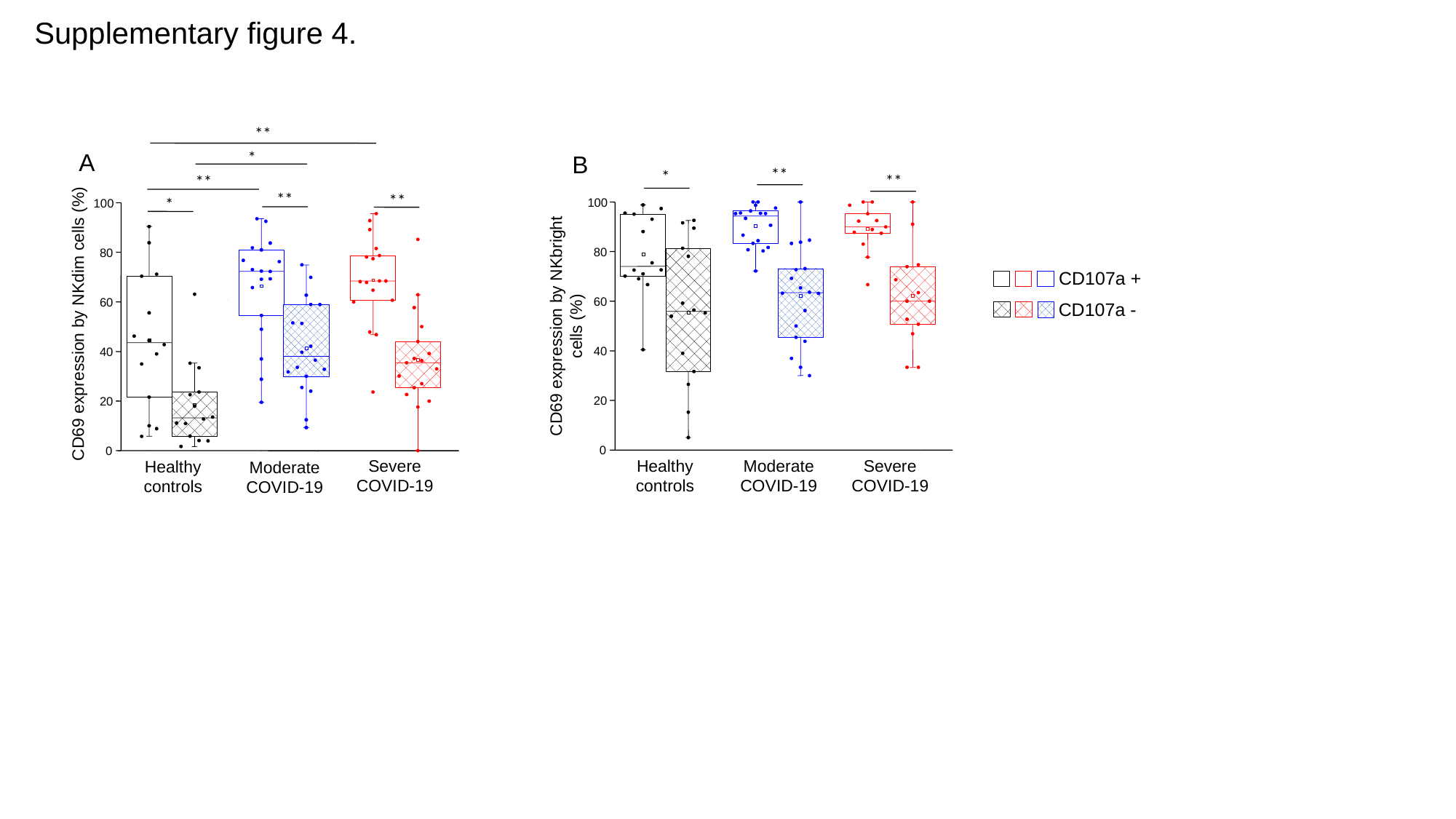

Supplementary figure 4.
**
*
**
**
Moderate
COVID-19
**
*
100
80
60
CD69 expression by NKdim cells (%)
40
20
0
Severe
COVID-19
Healthy controls
A
B
**
Moderate
COVID-19
*
**
Severe
COVID-19
100
80
60
CD69 expression by NKbright cells (%)
40
20
0
Healthy controls
CD107a +
CD107a -

## Slide 5
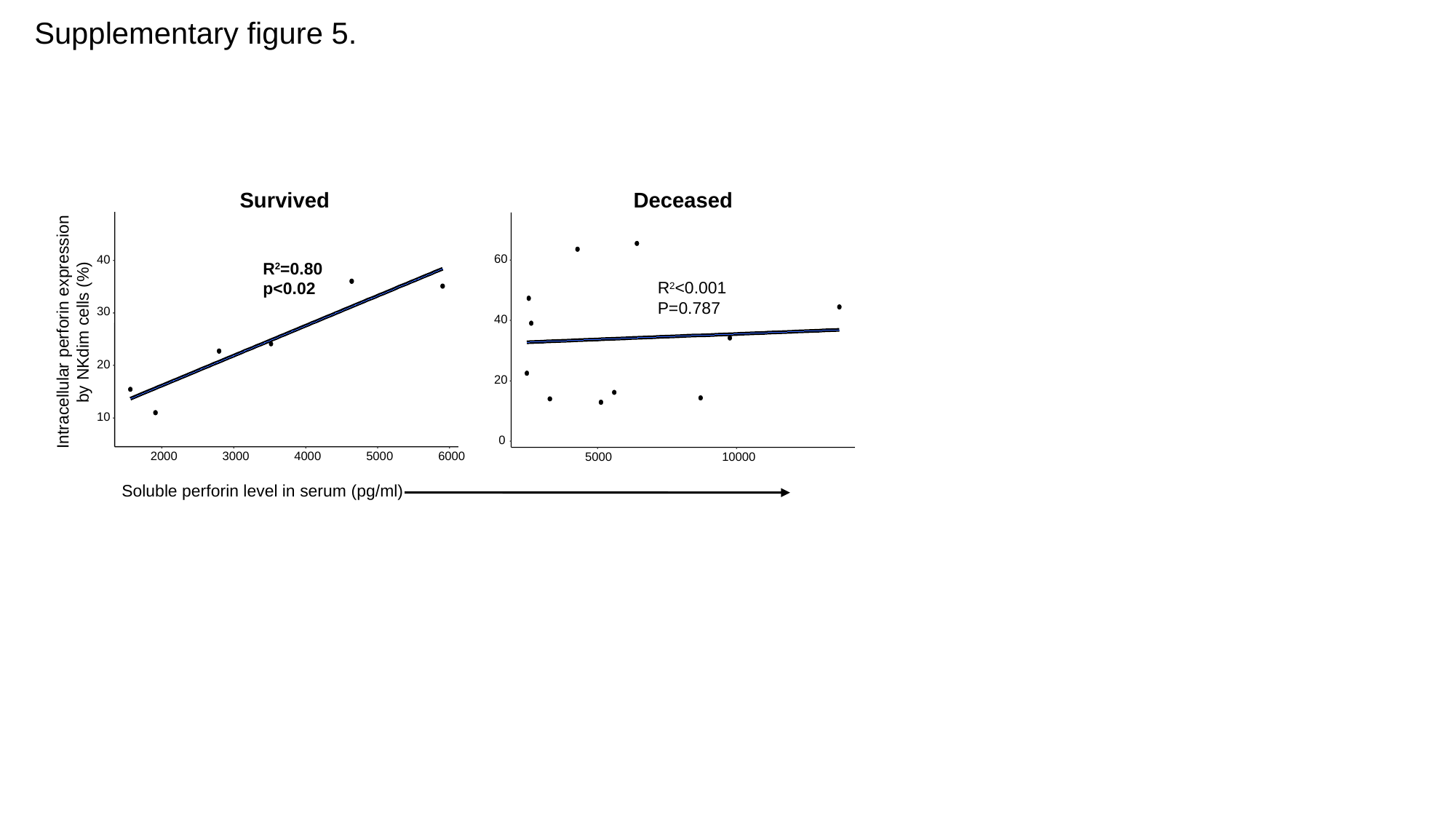

Supplementary figure 5.
Survived
40
R2=0.80
p<0.02
30
20
10
2000
3000
4000
5000
6000
Deceased
60
R2<0.001
P=0.787
40
20
0
5000
10000
Intracellular perforin expression by NKdim cells (%)
Soluble perforin level in serum (pg/ml)

## Slide 6
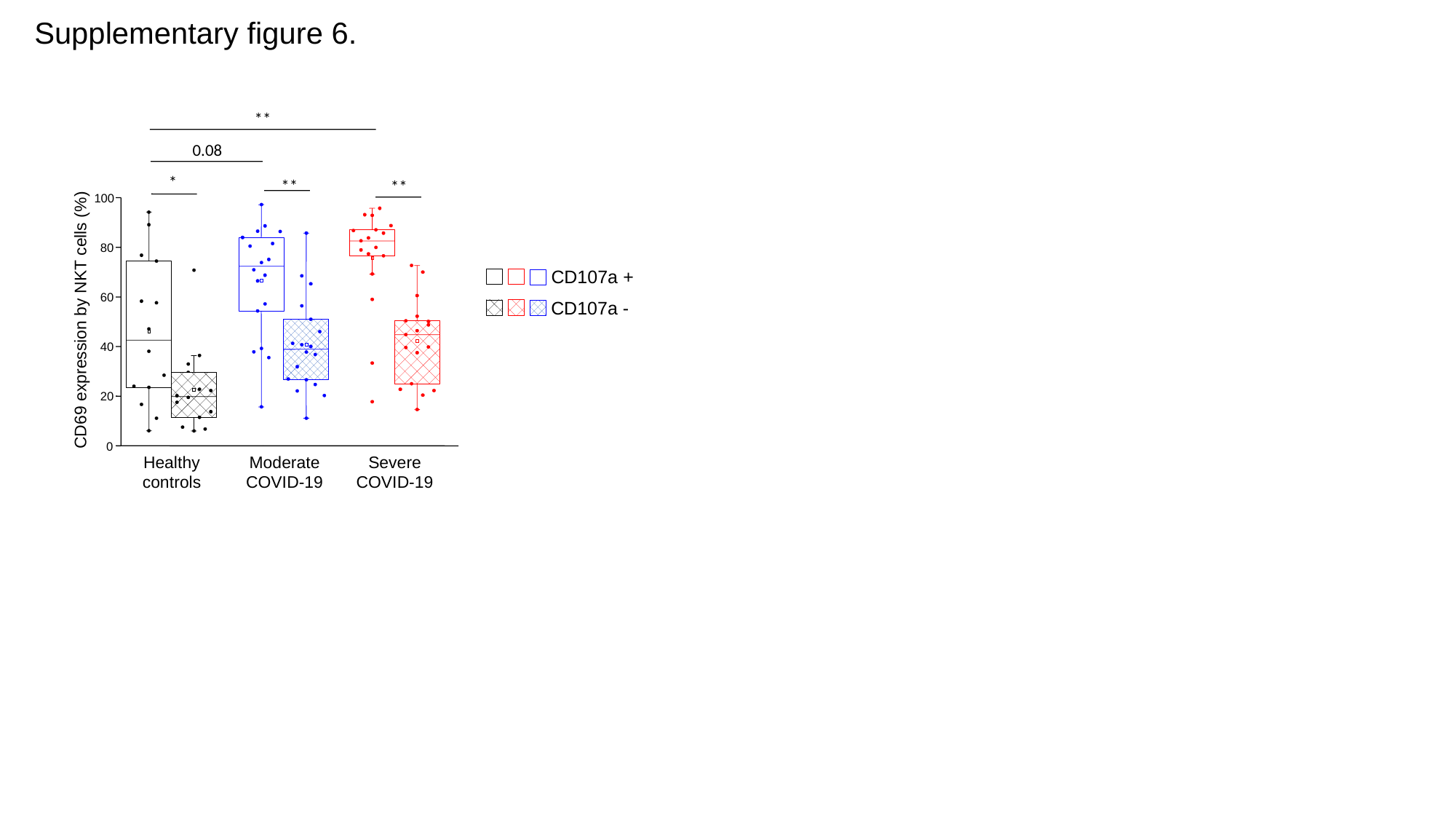

Supplementary figure 6.
**
0.08
*
**
**
Severe
COVID-19
100
Moderate
COVID-19
80
60
CD69 expression by NKT cells (%)
40
20
0
Healthy controls
CD107a +
CD107a -
